# Bioinformatic analysis of endometrial miRNA expression profile at day 26–28 of pregnancy in the mare

**DOI:** 10.1038/s41598-024-53499-x

**Published:** 2024-02-16

**Authors:** Agnieszka Sadowska, Tomasz Molcan, Anna Wójtowicz, Karolina Lukasik, Klaudia Pawlina-Tyszko, Artur Gurgul, Graca Ferreira-Dias, Dariusz J. Skarzynski, Anna Szóstek-Mioduchowska

**Affiliations:** 1 Department of Reproductive Immunology and Pathology, Institute of Animal Reproduction and Food Research of Polish Academy of Sciences, Tuwima Street 10, 10-748 Olsztyn, Poland; 2 Molecular Biology Laboratory, Institute of Animal Reproduction and Food Research, Polish Academy of Sciences, Tuwima Street 10, 10-748 Olsztyn, Poland; 3 Department of Animal Molecular Biology, National Research Institute of Animal Production, Sarego Street 2, 31-047 Kraków, Poland; 4 Center for Experimental and Innovative Medicine, University of Agriculture in Krakow, Mickiewicza Street 21, 31-120 Kraków, Poland; 5 CIISA-Center for Interdisciplinary Research in Animal Health, Faculty of Veterinary Medicine, University of Lisbon, 1300-477 Lisbon, Portugal; 6 Associate Laboratory for Animal and Veterinary Sciences (AL4AnimalS), 1300-477 Lisbon, Portugal

**Keywords:** Reproductive biology, Non-coding RNAs

## Abstract

The establishment of the fetomaternal interface depends on precisely regulated communication between the conceptus and the uterine environment. Recent evidence suggests that microRNAs (miRNAs) may play an important role in embryo-maternal dialogue. This study aimed to determine the expression profile of endometrial miRNAs during days 26–28 of equine pregnancy. Additionally, the study aimed to predict target genes for differentially expressed miRNAs (DEmiRs) and their potential role in embryo attachment, adhesion, and implantation. Using next-generation sequencing, we identified 81 DEmiRs between equine endometrium during the pre-attachment period of pregnancy (day 26–28) and endometrium during the mid-luteal phase of the estrous cycle (day 10–12). The identified DEmiRs appear to have a significant role in regulating the expression of genes that influence cell fate and properties, as well as endometrial receptivity formation. These miRNAs include eca-miR-21, eca-miR-126-3p, eca-miR-145, eca-miR-451, eca-miR-491-5p, members of the miR-200 family, and the miRNA-17-92 cluster. The target genes predicted for the identified DEmiRs are associated with ion channel activity and sphingolipid metabolism. Furthermore, it was noted that the expression of mucin 1 and leukemia inhibitory factor, genes potentially regulated by the identified DEmiRs, was up-regulated at day 26–28 of pregnancy. This suggests that miRNAs may play a role in regulating specific genes to create a favorable uterine environment that is necessary for proper attachment, adhesion, and implantation of the embryo in mares.

## Introduction

During the first month of equine pregnancy, there are various developmental and structural changes and events that occur in both the embryo and uterus. Many of these changes are unique to horses. Fertilization takes place in the fallopian tubes, but unlike other domestic animals, the equine embryo remains in the oviduct for an extended period, specifically 5.5 to 6.5 days. The embryo enters the uterus enclosed in a mucin-like glycoprotein capsule, which maintains the spherical shape of the equine embryo, a prerequisite for its mobility^1^. Prolonged mobility of the embryo within the uterine lumen (i.e. until approximately 15–16 days after ovulation) is crucial for maternal recognition of pregnancy, as well as for the establishment of a suitable uterine environment that supports the growth and development of the conceptus^2,3^. The equine embryo becomes 'fixed' in a single location of the uterine horn around day 16. However, it remains unattached to the endometrial luminal epithelium until day 40, when trophoblast microvilli begin to attach to the endometrial epithelium^4,5^. The embryo's lack of attachment and adherence to the endometrium is thought to be due to the mucin-like structure of the glycoproteins in the capsule surrounding the embryo, as well as the presence of anti-adhesive molecules (such as mucin 1; MUC1) on both the epithelium and the trophoblast^5^. From day 40 onwards, embryogenesis and organogenesis processes are followed by embryonic/fetal membrane differentiation and growth, endometrial cup formation, and the creation of the placenta. The placenta provides sustenance for the fetus to term at around 340 days of gestation^6^.

The establishment of the proper fetomaternal interface, early embryonic development, implantation, and maintenance of pregnancy depend on precisely regulated communication between the conceptus and the uterine environment. This communication is achieved through the secretion of steroid hormones, growth factors, cell adhesion molecules, and cytokines^7^. Numerous studies have shown that miRNAs play a role in regulating processes related to pregnancy recognition and embryo implantation in various domestic animal species (as reviewed by Bauersachs and Wolf, 2015^7^). miRNAs are a type of small, single-stranded non-coding RNA (18–22 nucleotides) that regulate gene expression by binding to a complementary mRNA target sequence, resulting in mRNA degradation or translation inhibition^8^. miRNAs have been demonstrated to play a role in regulating a range of physiological and pathological processes, such as cell proliferation, differentiation, adhesion, apoptosis, angiogenesis, reproduction, and tumorigenesis^9^. Several miRNAs, such as let-7g, miR-26a, miR-30, miR-34, miR-92b, miR-148, miR-214, miR-423, and miR-504, have been shown to regulate the expression of genes involved in human, bovine, porcine, ovine, goat, and rodent embryo implantation^10–16^. This includes the regulation of endometrial receptivity, angiogenesis, and immune tolerance^10–16^. In horses, miRNA expression profiles have been extensively studied, particularly during maternal recognition of pregnancy^2,17,18^. The identified miRNAs, including miR-19a, miR-101, miR-140, and miR-652, have been shown to regulate cell fate, inflammation, focal adhesion, as well as trophoblast proliferation, migration, and invasion. Based on these results it can be assumed a potential role of miRNAs in the embryo-maternal communication in mares. The previous study analyzed changes in the equine endometrial transcriptome, specifically on day 28 of pregnancy, which is around the time of embryo attachment^19^. The identified differentially expressed genes were related to prostaglandin (PG) production, progesterone synthesis, regulation of the innate immune system response, as well as cell proliferation, differentiation, and adhesion^19^. To the best of our knowledge, the expression profile, biological functions, and significance of miRNAs during this period of pregnancy in the mare remain unknown. Therefore, the primary objective of this study was to, using the next-generation sequencing (NGS), examine the alterations in miRNA expression pattern during the pre-implantation period of pregnancy (specifically, days 26–28). This investigation involved a comparative assessment with the miRNA expression profile in the cyclic endometrium during the post-ovulation period (days 10–12). Our goal was to identify differentially expressed miRNAs (DEmiRs) and predict their target genes, thereby shedding light on their potential roles within crucial cellular signaling pathways and biological processes essential for the successful implantation of the equine embryo. Furthermore, our study aimed to ascertain the expression levels of the DEmiR potential target genes (MUC1, LIF) being pivotal for the proper implantation of the equine embryo.

## Results

### The miRNA expression profile in equine endometrium

**Figure 1:**
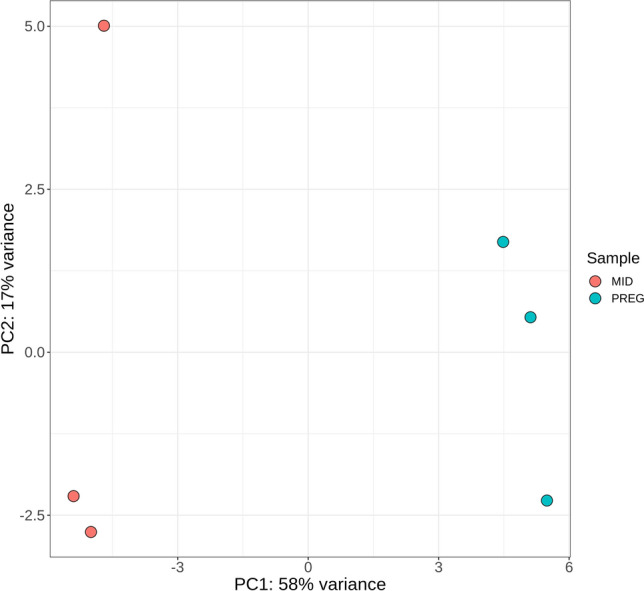
Graphical presentation of the first (PC1) and second (PC2) principal components (PCA) of the miRNA expression pattern of mare endometrium tissue. MID: mare endometrium samples obtained during mid-luteal phase of estrus cycle (Day 10–12); PREG: mare endometrium samples obtained during pre-attachment period of pregnancy (Day 26–28).

**Figure 2:**
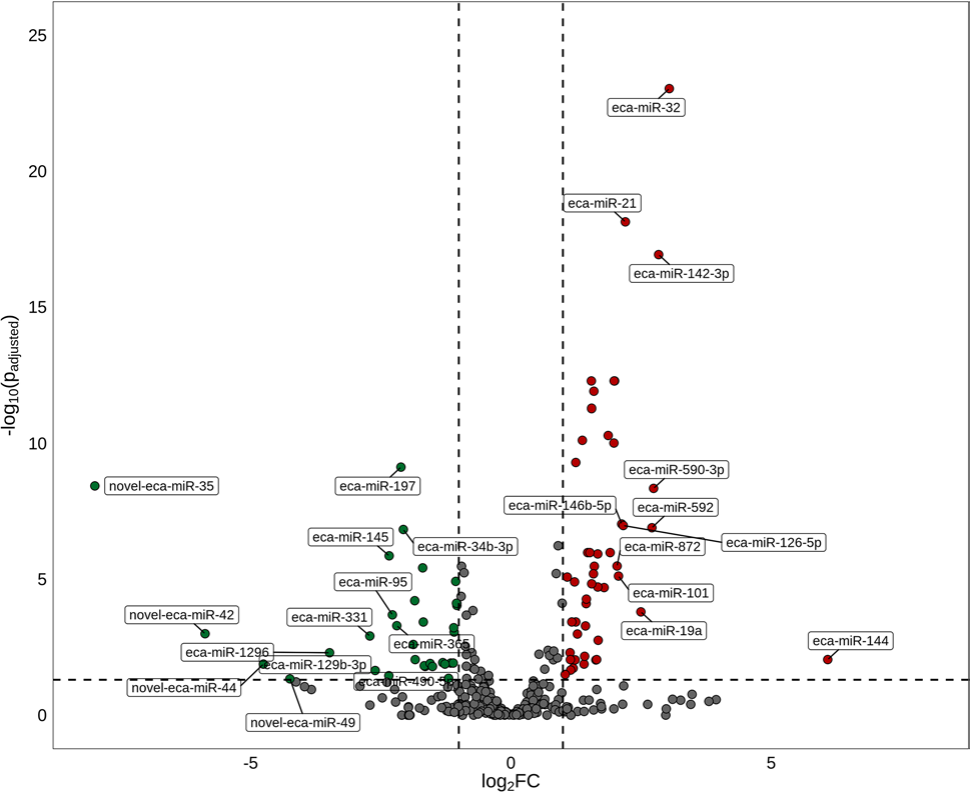
Volcano plot presenting all miRNAs, including differentially expressed miRNAs (DEmiRs; p_adjusted_ < 0.05, log2FC ≥ 1.0/log2FC ≤  − 1.0) identified in mare endometrium tissue. Please note that DEmiRs are represented by multicolored circles, where red color means up-regulated DEmiRs and green color depicts down-regulated DEmiRs. The grey circles represent all remaining miRNAs identified in the examined samples.

The sequencing of examined samples provided from 2 435 739 to 18 697 944 reads (42 NTs) per sample. The number of short-sequence reads obtained for each RNA sample is presented in Table 1. After rejecting low-quality reads (reads length < 18 and > 30 NTs, reads without a 3′-adapter or reads containing non-determined nucleotides “N”) and 3′-adapter sequences, the remaining reads (2.19–14.49 million reads per sample), were mapped to the sequences annotated as non-coding RNAs, except miRNAs, in the horse reference genome. The percentage contribution of specific ncRNA subtypes removed from the dataset is presented in Supplementary Table 1. Next, the remaining reads (1.96–10.49 million reads per sample) were mapped to the mature miRNA sequences in the miRBase database. The percentage of the aligned reads ranged from 47.9 to 65.2%, and an average of 98.9% of these reads were mapped to a unique (i.e., only one) location. The total number of mature miRNAs expressed in all examined samples ranged from 300 to 406 (Table 1), most of which were 21–23 NTs in length (88.44%), with the highest proportion being 22 NTs (48.59%), followed by 23 NTs (21.85%) and 21 NTs (18.00%; Supplementary Fig. 1, Supplementary Table 1). The PCA shows that the miRNAs identified in equine endometrium tissue during days 26–28 of pregnancy were clustered separately from those identified in endometrial tissue during the mid-luteal phase of the estrous cycle (Fig. 1). Volcano plot depicts the distribution of transcripts, including DEmiRs, identified in the examined samples (Fig. 2).

**Table 1:** Summary of sequence read alignments to the reference genome.

Total number of	MID_1	MID_2	MID_3	PREG_1	PREG_2	PREG_3
Reads before QC	13,143,264	18,697,944	10,464,675	6,216,685	3,290,878	2,435,739
Reads after QC	11,044,633	14,493,659	8,725,964	5,593,764	2,963,613	2,199,563
Reads after removing ncRNA, other than miRNA	9,532,401	10,494,950	6,235,201	5,118,431	2,698,099	1,958,293
Mapped reads	5,180,594	5,303,608	2,987,504	3,336,187	1,662,435	1,194,826
Uniquely mapped reads	5,115,562	5,240,277	2,948,815	3,310,592	1,646,619	1,181,721
Expressed known miRNAs	320	329	323	287	280	284
Expressed novel miRNAs	59	77	53	39	20	26

QC: quality control; MID: mare endometrium samples obtained during mid-luteal phase of estrus cycle (day 10–12); PREG: mare endometrium samples obtained during pre-attachment period of pregnancy (day 26–28); _1, _2, _3: biological replicates.

**Figure 3:**
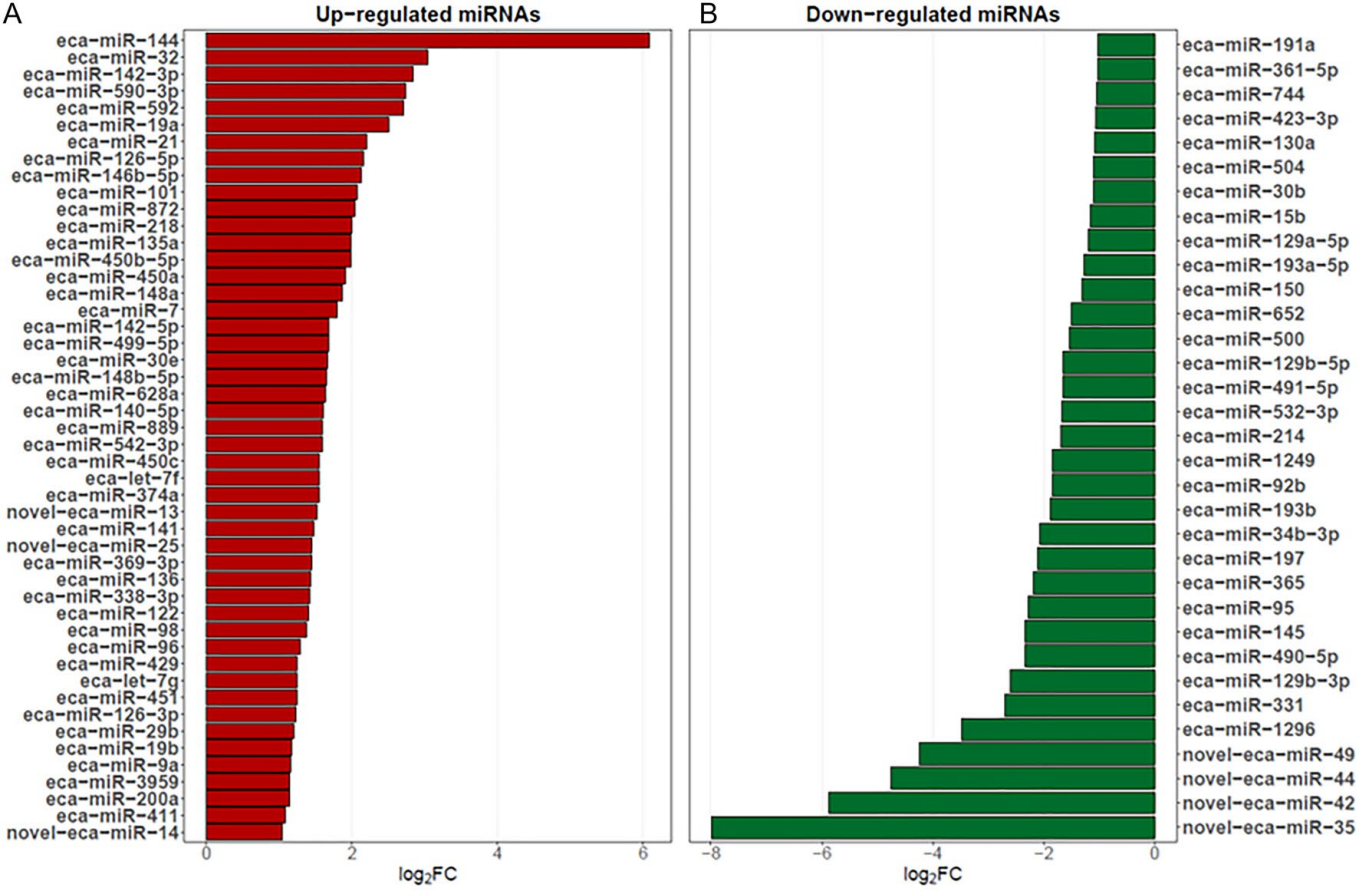
Diagram presenting differentially expressed miRNAs (DEmiRs; p_adjusted_ < 0.05, log2FC ≥ 1.0/log2FC ≤  − 1.0) identified in mare endometrium tissue during pre-attachment period of pregnancy (day 26–28).

**Figure 4:**
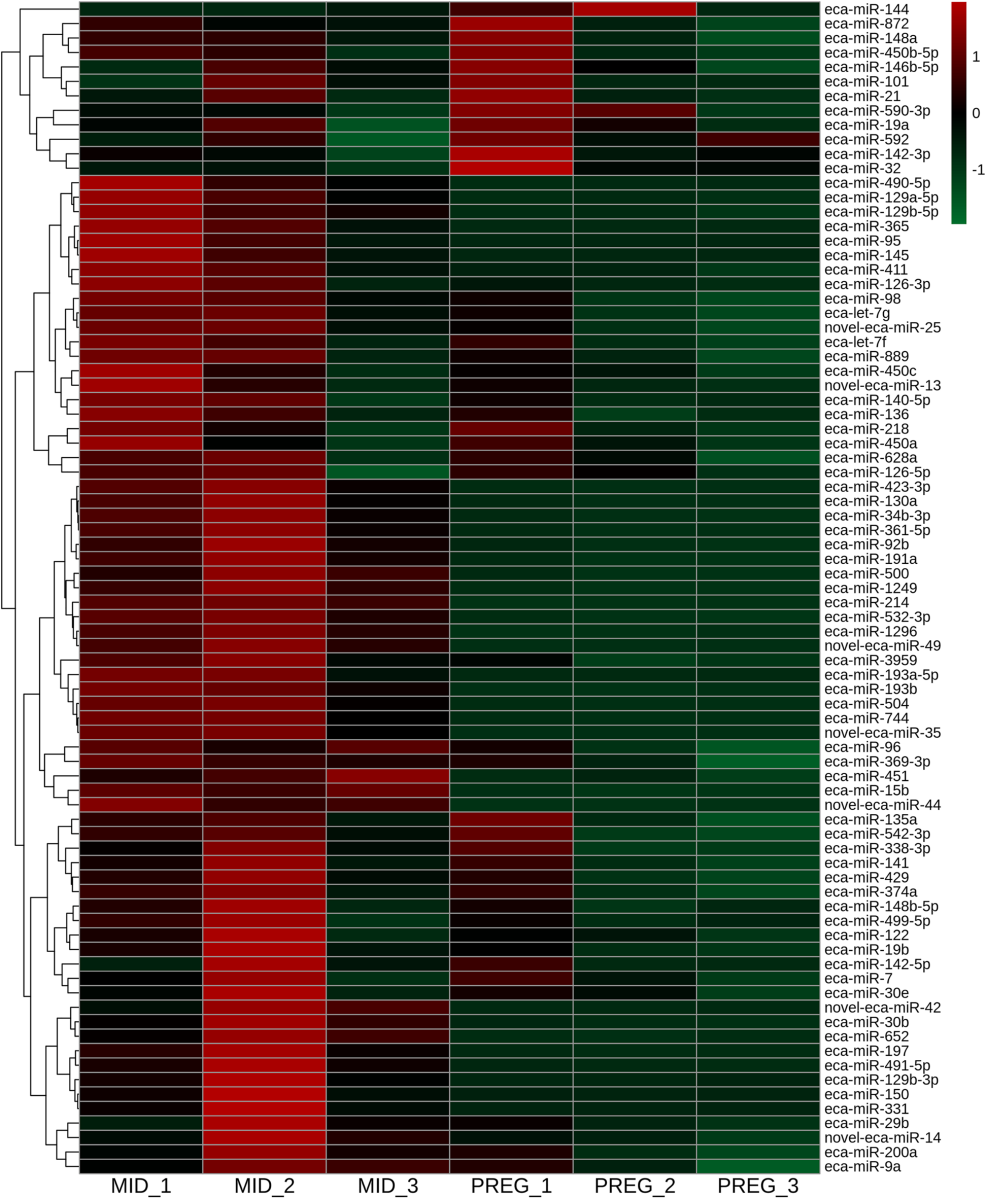
Heatmap illustrating the expression profile of the differentially expressed miRNAs (DEmiRs; p_adjusted_ < 0.05, log2FC ≥ 1.0/log2FC ≤  − 1.0) identified in mare endometrium tissue. The color scale of the heatmap shows the normalized (Z-score) expression level where red blocks represent up-regulated DEmiRs, and green blocks represent down-regulated DEmiRs. MID: mare endometrium samples obtained during mid-luteal phase of estrus cycle (day 10–12); PREG: mare endometrium samples obtained during pre-attachment period of pregnancy (day 26–28); _1, _2, _3: biological replicates.

The expression of 81 miRNAs, including seven novel miRNAs, differed significantly (DEmiRs; p_adjusted_ < 0.05, log2FC ≥ 1.0/log2FC ≤  − 1.0; Supplementary Table 3) between the examined endometrial tissues. We identified 48 up-regulated and 33 down-regulated DEmiRs (Supplementary Table 3 and Fig. 3). The changes in the expression levels of the up-regulated and down-regulated DEmiRs are visualized in the heatmap (Fig. 4).

**Figure 5:**
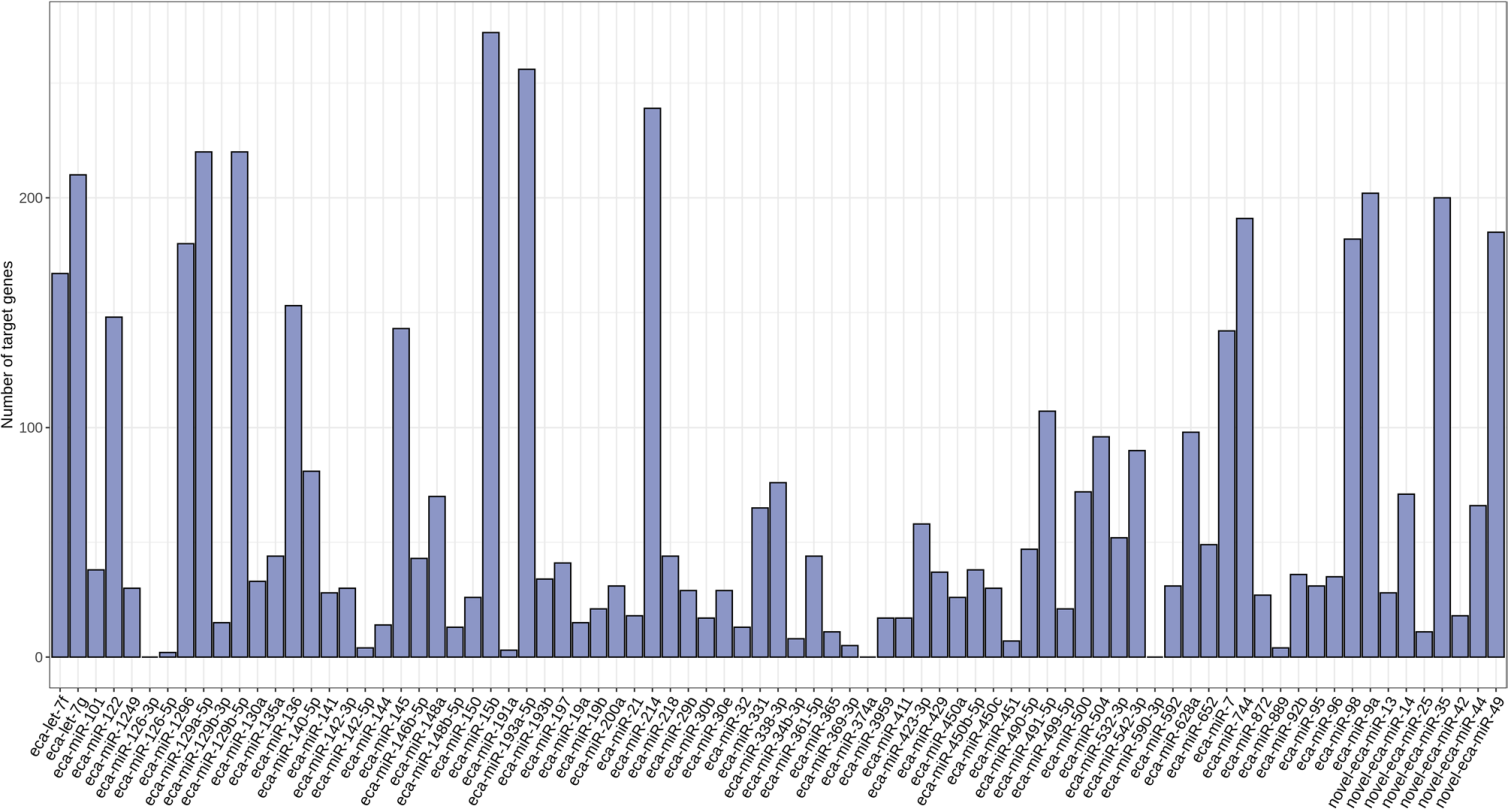
Diagram presenting number of target genes predicted for differentially expressed miRNAs (p_adjusted_ < 0.05, log2FC ≥ 1.0/log2FC ≤  − 1.0) identified in mare endometrium tissue during pre-attachment period of pregnancy (day 26–28).

A total of 4460 target genes were predicted for the 81 identified DEmiRs. The number of predicted target genes ranged from 2 for eca-miR-126-5p to 272 for eca-miR-15b (Fig. 5 and Supplementary Table 4). No targets were identified for three DEmiRs: eca-miR-126-3p, eca-miR-374a, and eca-miR-590-3p.

### Functional classification of DEmiR target genes

**Figure 6:**
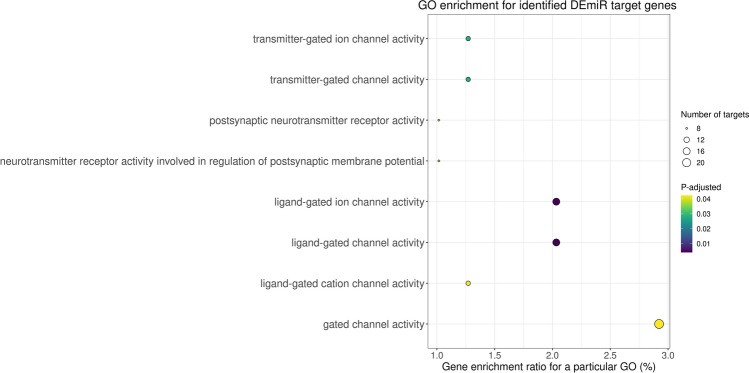
Dot plot illustrating Gene Ontology (GO) pathway enrichment analysis of the target genes predicted for differentially expressed miRNAs (DEmiRs; p_adjusted_ < 0.05, log2FC ≥ 1.0/log2FC ≤  − 1.0) identified in mare endometrium tissue during pre-attachment period of pregnancy (day 26–28). The size of dots depends on number of target genes assigned to particular processes, while the dot color depends on pathway enrichment significance.

**Figure 7:**
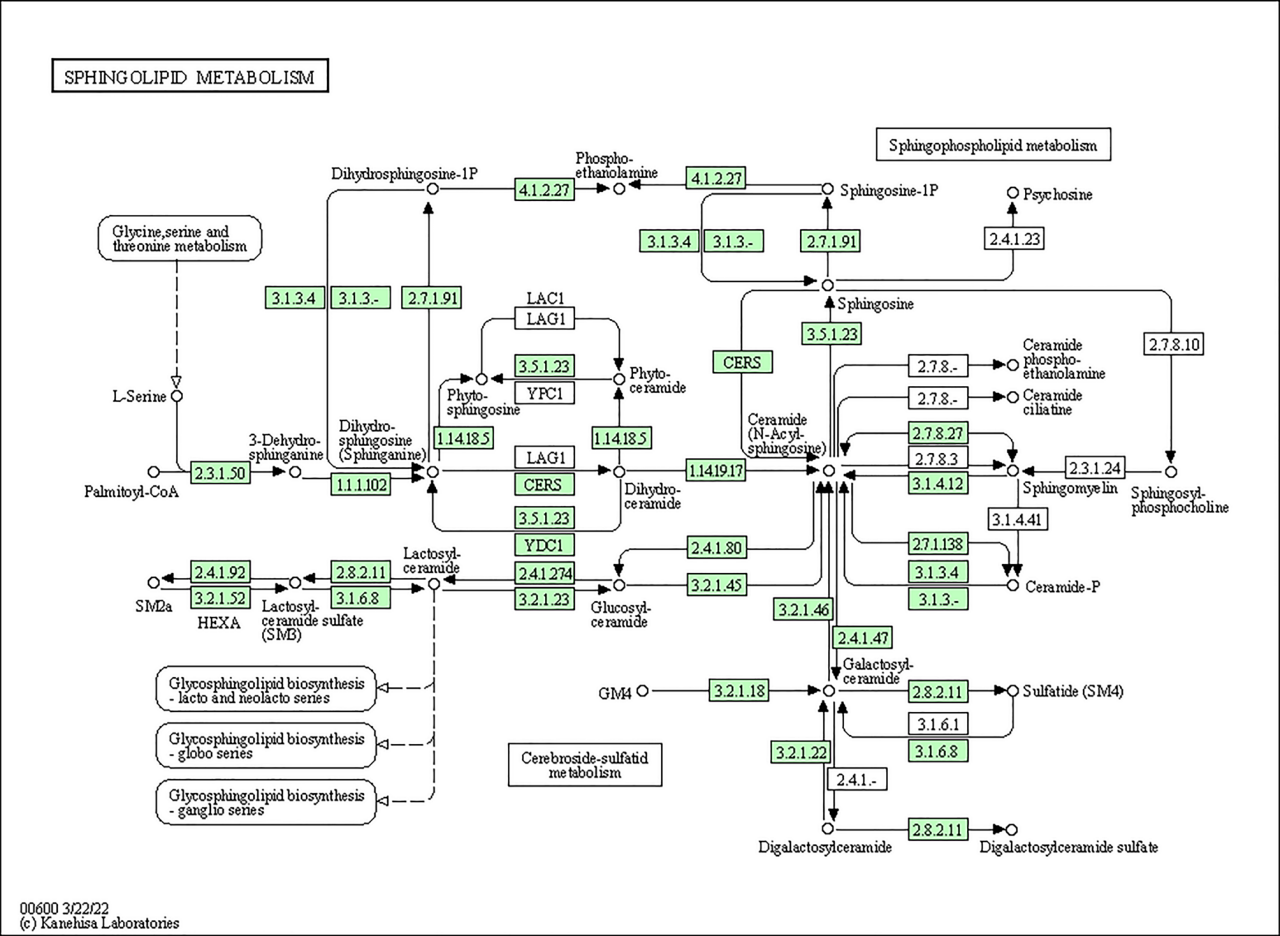
KEGG analysis of target genes predicted for differentially expressed miRNAs (DEmiRs; p_adjusted_ < 0.05, log2FC ≥ 1.0/log2FC ≤  − 1.0) identified in mare endometrium tissue during pre-attachment period of pregnancy (day 26–28), and assigned to the sphingolipid metabolism pathway. The target genes predicted for DEmiRs are marked in green.

To indicate the possible role of the miRNAs identified in the current study, a functional analysis of DEmiR target genes was performed. These genes were then classified into the 'molecular function' category of GO terms, where they were found to be primarily associated with ligand-gated channel activity, transmitter-gated ion channel activity, and neurotransmitter receptor activity (Fig. 6 and Table 2). Notably, none of the DEmiR target genes were assigned to either the 'biological process' or 'cellular components' categories. The KEGG database was used to perform a subsequent functional classification of all DEmiR target genes. In this study, 17 of the identified DEmiR target genes were assigned to only one signaling pathway, which is sphingolipid metabolism (Fig. 7 and Table 3).

**Table 2:** Gene Ontology (GO) enrichment analysis of target genes predicted for differentially expressed miRNAs (p_adjusted_ < 0.05, log2FC ≥ 1.0/log2FC ≤  − 1.0) in mare endometrium samples obtained during pre-attachment period of pregnancy (day 26–28).

No	GO term ID	Term name	Term category	p_adjusted_	Number of miRNA target genes enriched to GO term	Number of miRNA target genes enriched to particular GO term	List of miRNA target genes enriched to the particular GO term
1	GO:0015276	Ligand-gated ion channel activity	Molecular function	0.00421618620809567	787	16	CHRNA6, CHRNB3, KCNJ16, ASIC2, KCNJ13, GABRE, GABRG1, GLRA4, HTR3A, GABRB3, GLRA1, KCNJ5, ASIC3, CFTR, CHRND, CHRNA1
2	GO:0022834	Ligand-gated channel activity	molecular function	0.00421618620809567	787	16	CHRNA6, CHRNB3, KCNJ16, ASIC2, KCNJ13, GABRE, GABRG1, GLRA4, HTR3A, GABRB3, GLRA1, KCNJ5, ASIC3, CFTR, CHRND, CHRNA1
3	GO:0022824	Transmitter-gated ion channel activity	Molecular function	0.0281591760051717	787	10	CHRNA6, CHRNB3, GABRE, GABRG1, GLRA4, HTR3A, GABRB3, GLRA1, CHRND, CHRNA1
4	GO:0022835	Transmitter-gated channel activity	Molecular function	0.0281591760051717	787	10	CHRNA6, CHRNB3, GABRE, GABRG1, GLRA4, HTR3A, GABRB3, GLRA1, CHRND, CHRNA1
5	GO:0022836	Gated channel activity	Molecular function	0.0422960252917783	787	23	CHRNA6, CHRNB3, VDAC3, KCNJ16, CACNB1, ASIC2, KCNJ13, GABRE, GABRG1, GLRA4, CACNG7, KCNV2, TMEM150C, HTR3A, GABRB3, GLRA1, SNAP25, KCNJ5, KCNC4, ASIC3, CFTR, CHRND, CHRNA1
6	GO:0098960	Postsynaptic neurotransmitter receptor activity	Molecular function	0.0422960252917783	787	8	CHRNA6, CHRNB3, CHRM1, GABRG1, GABRB3, GLRA1, CHRND, CHRNA1
7	GO:0099529	Neurotransmitter receptor activity involved in regulation of postsynaptic membrane potential	Molecular function	0.0422960252917783	787	8	CHRNA6, CHRNB3, CHRM1, GABRG1, GABRB3, GLRA1, CHRND, CHRNA1
8	GO:0099094	Ligand-gated cation channel activity	molecular function	0.0422960252917783	787	10	CHRNA6, CHRNB3, KCNJ16, ASIC2, KCNJ13, HTR3A, KCNJ5, ASIC3, CHRND, CHRNA1

**Table 3:** KEGG enrichment analysis of target genes predicted for differentially expressed miRNAs (p_adjusted_ < 0.05, log2FC ≥ 1.0/log2FC ≤  − 1.0) in mare endometrium samples obtained during pre-attachment period of pregnancy (day 26–28).

No.	KEGG ID	KEGG name	p_adjusted_	Number of miRNA target genes enriched to particular KEGG	List of miRNA target genes enriched to the particular KEGG
1	ecb00600	Sphingolipid metabolism	0.0307358392389343	17	ASAH2, CERS5, HEXB, GLB1, ACER2, GLA, GAL3ST1, KDSR, SPTLC1, PSAP, ACER3, SGPL1, CERK, B4GALNT1, PLPP1, GALC, LOC111770943

### Validation of selected DEmiRs by RT-qPCR

**Figure 8:**
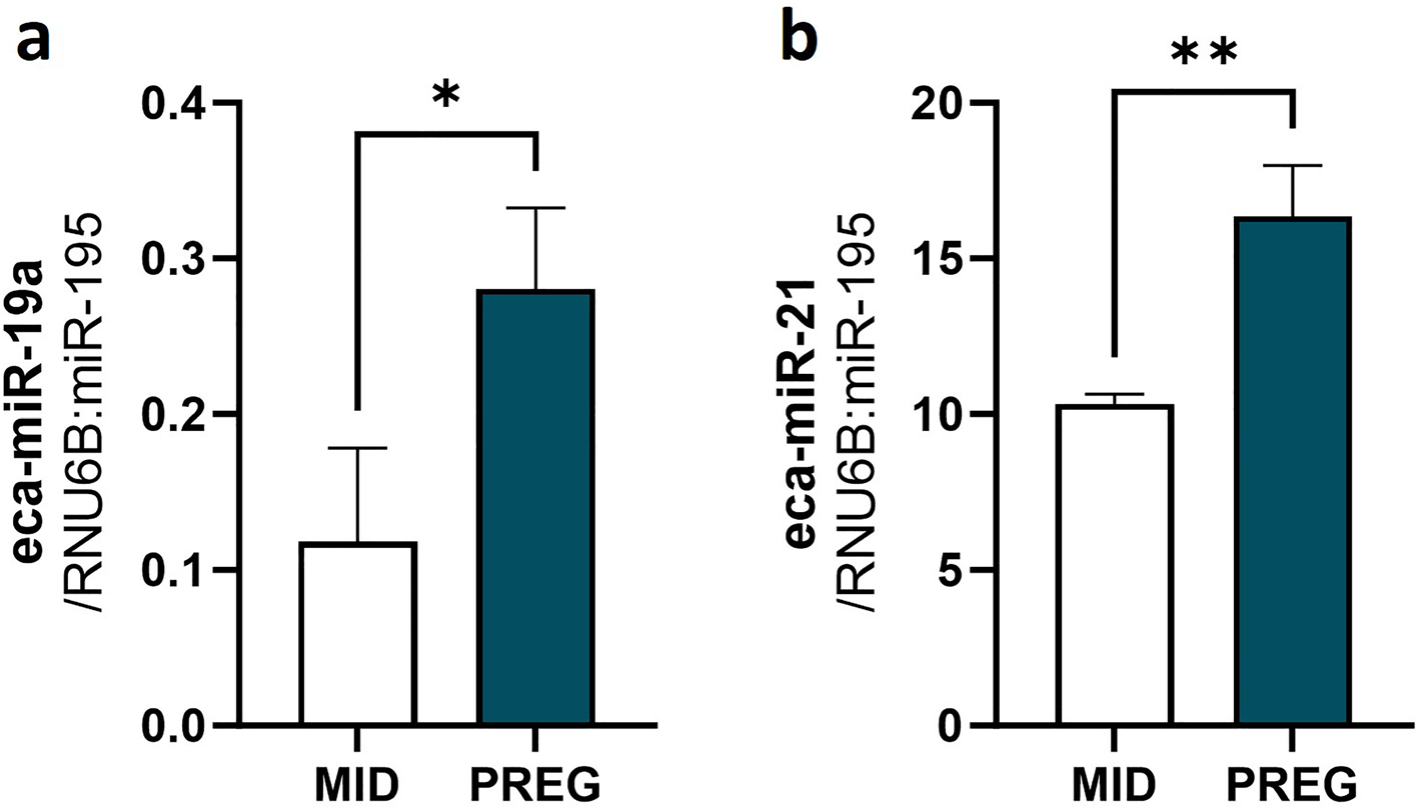
Real-time validation of the selected differentially expressed miRNAs (DEmiRs; **a** eca-miR-19a; **b** eca-miR-21) identified in mare endometrium tissue during preimplantation period of pregnancy by RNA-Seq. The validation was performed using the same RNA samples as used in the NGS. Data were expressed as mean ± SD. Statistical analysis was performed using Student’s t-test. Different superscripts designate statistical differences (p < 0.05). MID: mare endometrium samples obtained during mid-luteal phase of estrus cycle; PREG: mare endometrium samples obtained during pre-attachment period of pregnancy (day 26–28).

To validate the NGS results, we selected two miRNAs, namely eca-miR-19a and eca-miR-21, for RT-qPCR. The expression of eca-miR-19a (Fig. 8a) and eca-miR-21 (Fig. 8b) confirmed the NGS results.

### Verification of the expression of genes being potential targets of DEmiRs

**Figure 9:**
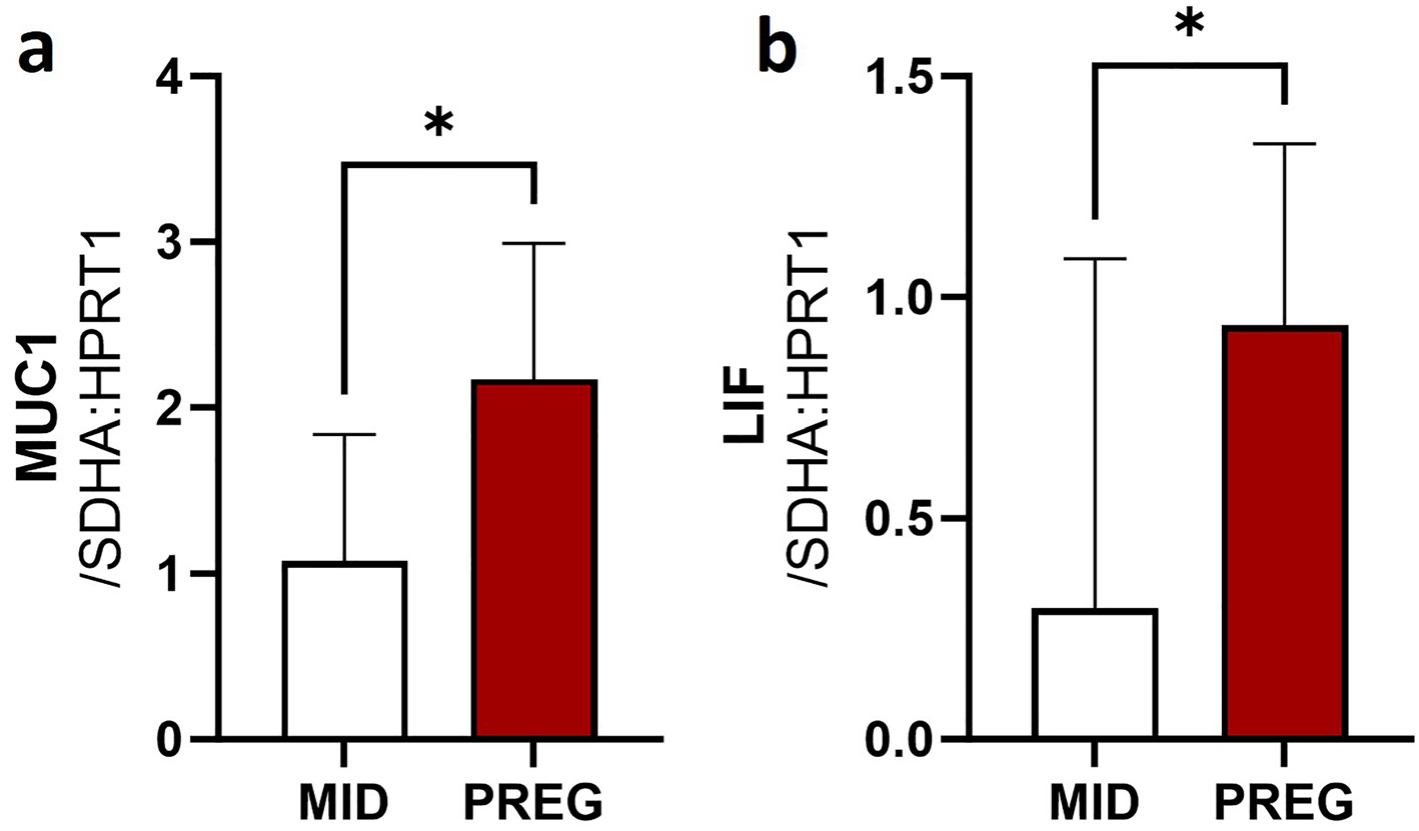
Real-time determination of expression of (**a**) MUC1 and (**b**) LIF, genes potentially regulated by identified in mare endometrium tissue during pre-attachment period of pregnancy (Day 26–28) DEmiRs. Data were expressed as mean ± SD. Statistical analysis was performed using Student’s t-test. Different superscripts designate statistical differences (p < 0.05). MID: mare endometrium samples obtained during mid-luteal phase of estrus cycle (day 10–12); PREG: mare endometrium samples obtained during pre-attachment period of pregnancy (day 26–28).

The expression of MUC1, which is a potential target of eca-miR-145, and LIF, which is a potential target of eca-miR-34b, eca-miR-423, eca-miR-491-5p, and eca-miR-500, was found to be up-regulated during day 26–28 of pregnancy compared to the mid-luteal phase of the estrus cycle (Fig. 9).

## Discussion

microRNAs have been widely reported to be involved in embryo-uterine cross-talk during the preimplantation period of pregnancy in multiple species, including primates, rodents, and pigs^20^. In this study, 81, including 48 up-regulated and 33 down-regulated DEmiRs were identified in the equine endometrium during the pre-attachment period of pregnancy compared to the mid-luteal phase of the estrus cycle. A total of 4460 targets were predicted for the identified DEmiRs. These targets were mainly involved in sphingolipid metabolism, as well as ligand-gated channel activity, transmitter-gated ion channel activity, and neurotransmitter receptor activity. These processes are important for creating a favorable uterine environment for proper embryo attachment, adhesion and implantation.

Sphingolipids, vital components of the cell membrane, play an important role in regulating cellular fate in reproductive processes, particularly during pregnancy^21^. Disruptions in sphingolipid metabolism can impair uterine blood vessel formation and cause early pregnancy loss in mice^22^. Furthermore, sphingolipids regulate the expression of actin-binding proteins (ERM protein family), affecting cell adhesion, which is crucial for embryo-uterine interaction^23,24^. Data concerning the involvement of miRNAs in regulating the expression of enzymes associated with sphingolipid biosynthetic pathways are limited. Results of the studies carried out mostly in cancer cell lines, demonstrated that miR-9, miR-29b, and miR-101 regulate the expression of enzymes involved in the biosynthesis of sphingolipids, including ceramide^25–27^. However, as far as we know, no study has yet demonstrated the expression of genes associated with sphingolipid metabolism and their regulation by miRNA in the mare endometrium during early pregnancy. Nevertheless, further research is required to fully investigate this matter, based on findings in various species and the results of our investigation.

Ion channels are vital membrane proteins that facilitate the transfer of ions across the cell or organelle membrane, resulting in changes in membrane potential, ion gradients, pH, and second messenger signaling^28–31^. Their expression was found in the human, rodent, and porcine endometrium^28–31^. Additionally, Cl−, K+, and Na+ channels have been recently detected in the endometrium of pregnant mares^19^. In the uterus, ion channels primarily regulate the volume and composition of the electrolyte and water-based fluid. A reduction in the volume of uterine fluid leads to the closure of the uterine lumen, preventing the embryo from moving and facilitating its implantation^32^. It was demonstrated that ion channels present in the endometrium regulate endometrial epithelial cell proliferation and apoptosis, endometrial prostaglandin synthesis, and protease activity^32^. In humans and rodents, abnormal expression or activity of endometrial ion channels resulted in implantation failure^32^. Currently, in the literature, there is a lack of information regarding the involvement of miRNA molecules in regulating the expression and activity of uterine ion channels during embryo implantation. Previous research has primarily focused on cardiovascular (miR-21, miR-29b) and nervous systems (miR-9, miR-30b, miR-92, miR-129, miR-142), as well as cancer cell lines (miR-34), as models^33–35^. In conclusion, miRNAs may play an important role in embryo-uterine interaction in mares by regulating the expression of genes related to ion channel activity, thus preparing a suitable uterine environment.

Successful embryo implantation requires a receptive endometrium. The endometrial stromal and epithelial cells proliferate and differentiate to form a receptive endometrium, which allows for the attachment, adhesion and implantation of an embryo^7,36^. Results of our study, along with other studies conducted on humans, rodents, pigs and goats have indicated that several miRNAs, including let-7g, miR-21, miR-30e, miR-96, miR-126-3p, miR-135a, miR-145, miR-338-3p, miR-451, miR-490-5p, miR-504, as well as members of the miR-200 family (miR-200a, miR-141, miR-429) and miR-17-92 clusters (miR-19a, miR-19b), are differentially expressed in the receptive endometrium^37–40^. These miRNAs were shown to target KLF-12^41^, PTEN^42^, integrin α-11^43^ and ZEB^44^ as well as genes involved in signaling pathways including the canonical Wnt signaling pathway^45^, NF-kB, P13K/AKT, NOTCH1, ECM-receptor-interaction, STAT3/HIF, p38/MAPK^46^ and TGF-β signaling pathway^37^. All those genes are known for their involvement in the regulation of cellular adhesion, migration, proliferation, and differentiation, as well as interactions between cells and extracellular matrix proteins. Based on these observations, the above-mentioned miRNAs seem to play a crucial role in preparing the uterine endometrium for equine embryo attachment, adhesion, and implantation by regulating the expression of specific genes. However, further studies are required to investigate their precise role in establishing endometrial receptivity in mares to explore its exact role during endometrial receptivity establishment in the mare.

The results of the current study demonstrated that the expression of eca-miR-145 was reduced in the endometrium of pregnant mares. miR-145 has been shown to be a critical regulator during embryo attachment, adhesion and implantation in humans and rats^47–50^. It regulates the expression of several genes, such as IGF1R, COX-2, MMP-9, MMP-11, VEGF, HO-1, sFRP, and FSCN-1, which are recognized for their pro-angiogenic and anti-inflammatory properties, as well as their role in modulating cell adhesion and cell–cell interactions^37,47–50^. Additionally, miR-145 targets MUC1^50^, which expression was found to be up-regulated in the current study. Mucin 1 is a transmembrane glycoprotein that provides lubrication, hydration, and protection against external pathogens in the endometrium. It is present in the luminal and glandular epithelium of various mammalian species, including horses, as well as in horse placental tissue^5^. In many mammalian species, the removal or downregulation of MUC1, which creates an anti-adhesive barrier that is crucial for proper embryo adherence and implantation, occurs. However, this does not seem to be the case in horses^51^. The equine embryo strategy for adhesion to endometrial epithelium appears to differ from that of other species. Despite being 'fixed' at the base of a uterine horn from about day 16, the equine embryo remains unattached to the endometrial luminal epithelium until day 40^52^. According to Wilsher and colleagues^5^, MUC1 protein is present at the embryo-maternal interface during different stages of equine pregnancy, including the pre-attachment stage (days 20–37). They concluded that equine embryo implantation occurs regardless of the presence of MUC1. However, there are indications that, despite its well-known anti-adhesive properties, MUC1 may also facilitate cell adhesion. The structure of MUC1 oligosaccharides can change to enable binding with the trophoblast. Therefore, certain MUC1 glycoforms, such as those bearing LNF-1 or selectin ligands, may have pro-adhesive properties^53^. Hey and Aplin^54^ reported that MUC1 can bind intercellular adhesion molecules, such as SLex and Slea. However, the exact mechanisms by which this protein may influence embryo adhesion and implantation in the mare remain unclear due to conflicting reports of its pro- and anti-adhesive properties. Further research is required to determine the specific role of MUC1 during the pre-attachment period of equine pregnancy, as well as the role of miR-145, which regulates its expression.

The present study found that the eca-miR-34b, eca-miR-423, eca-miR-491-5p and eca-miR-500 expression was downregulated in pregnant mares. These miRNAs were previously shown to regulate the expression of LIF^55–58^, which was found to be up-regulated in pregnant endometrium in the current study. An increase in the expression of LIF and its receptor has been observed during the peri-implantation period of pregnancy, particularly during the establishment of uterine receptivity, in various species, including horses^19,59,60^. LIF may enhance embryo attachment, adhesion and implantation through various mechanisms. LIF activates the JAK-STAT3 pathway, influencing uterine physiology, including cell fate, angiogenesis, and innate immune response^61^. Additionally, LIF promotes the expression of miR-21 via STAT3 activation, facilitating epithelial-mesenchymal transition crucial for embryo implantation^62^. LIF also stimulates the production of MMP-9 and uPA, enzymes involved in tissue remodeling and angiogenesis^63^. While LIF appears to be a shared target for multiple miRNAs during equine endometrial receptivity formation, further research is essential to deepen our comprehension of LIF and the associated miRNA regulatory pathways during embryo attachment and implantation in the mare.

In conclusion, it appears that the identified in the current study miRNAs may influence the expression of numerous genes, including those involved in the ion channel activity, sphingolipid metabolism, formation of endometrial receptivity, angiogenesis, and ECM remodeling. Our results suggest that the identified miRNAs may be important for successful embryo attachment, adhesion and implantation through the preparation of a suitable uterine environment in mares. Identifying significant miRNAs in the pregnant equine endometrium is crucial for understanding the role of these molecules during the pre-attachment period of pregnancy. This knowledge may help reduce the rate of pregnancy loss in the future. However, more detailed research is required to specify the role of individual miRNAs during early pregnancy in the mare.

## Material and methods

### Animal study and tissue collection

Endometrial tissue from six clinically healthy, normally cycling, multiparous, mixed breed mares (aged 3–6 years, weighing 500 ± 100 kg) was used in this study. The procedures were reviewed and approved by the Local Ethics Committee for Experiments on Animals in Olsztyn, Poland (approval number 51/2011). The study was carried out between April and June 2016. The mares were housed in private stables with ad libitum access to water and fed hay and cereals. The horses were considered to be healthy based on a physical examination by a veterinarian. The animals also had no reproductive tract abnormalities, which was confirmed by ultrasonography. In addition, the endometrium was of category I according to Kenney and Doig^64^ as assessed by microscopic observation after hematoxylin–eosin staining. Prior to the experiment, mares received two doses of a PGF2α analog (5 mg dinoprost; Dinolytic, Zoetis, Poland) 12 days apart to synchronize estrus. Follicular development was monitored in the mares by transrectal ultrasonography (USG) using a 7.5 MHz linear probe (MyLabOne Vet Ultrasound System; ESOATE Pie Medica, Genoa, Italy) at 12-h intervals during the periovulatory period until ovulation. In addition, visible signs of estrus (i.e., vaginal mucus and standing behavior) and structural changes in the corpus luteum were assessed by USG every 2 days until day 10 (day 0 = ovulation day). Three mares were inseminated by natural mating with the same stallion on day 0 of the estrous cycle. The day after mating was identified as the first day of pregnancy. Pregnancy was determined by USG and additionally confirmed by embryo flashing/collection from the uterus during slaughter. Uteri were collected from mares at a local abattoir on days 10–12 of the estrous cycle (control group; non-inseminated mares; n = 3) and days 26–28 of pregnancy (inseminated mares; n = 3). Endometrial samples were collected 5–10 min post-slaughter, transferred to RNAlater (Invitrogen, Carlsbad, California, USA), transported to the laboratory at 4 °C and immediately processed for RNA isolation and NGS. Animals were slaughtered for meat as part of routine breeding as slaughter animals.

### miRNA isolation

For NGS, total RNA was extracted using the Direct-zol RNA MiniPrep Kit (Zymo Research, Irvine, California, USA) according to the manufacturer's protocol. RNA concentration and quality were measured using a NanoDrop 2000 spectrophotometer (Thermo Fisher Scientific, Waltham, Massachusetts, USA) and a 2200 TapeStation instrument (Agilent, Santa Clara, USA); only samples with an A260 nm/230 nm ratio between 1.8 and 2.2 and an RNA integrity number greater than 7.5 were used.

### miRNA sequencing

miRNA sequencing was performed as previously described^65^. Briefly, miRNA libraries were prepared using the NEBNext Multiplex Small RNA Library Prep Set for Illumina (New England Biolabs, Ipswich, MA, USA) according to the manufacturer's instructions. Specifically, after 3′ adapter ligation, hybridization of the reverse transcription primer and 5′ adapter ligation, reverse transcription and PCR amplification of the resulting products were performed. The PCR was performed using 12 different indexed primers containing a unique sequence of 6 NTs in length, allowing barcoding of each library and multiplexing of samples during sequencing. The next step was the size selection (Novex 6% TBE PAGE gel, [Invitrogen]) of the libraries. The quantity of the obtained libraries was then measured using a Qubit 2.0 Fluorometer (Thermo Fisher Scientific), while a 2200 TapeStation instrument (Agilent Technologies) was used to assess their size. The obtained libraries were then sequenced on a HiScanSQ sequencing instrument (Illumina, San Diego, CA, USA) according to the manufacturer's protocol.

### Bioinformatic analysis

The quality of raw reads obtained after sequencing was evaluated using FastQC (https://www.bioinformatics.babraham.ac.uk/projects/fastqc/^66^). Next, 3′-adapter sequences as well as reads shorter than 18 NTs or longer than 30 NTs, reads without a 3′-adapter or containing non-determined nucleotides “N” were removed from the dataset by Cutadapt software (v2.8^67^). The next step included the removal of transcripts mapped to the sequences annotated as non-coding RNAs, except miRNAs, in the horse reference genome (Equus_caballus.EquCab3.0.105, Ensembl database, release 105; Bowtie2 software, v2.4.2^68^). The remaining reads were subsequently mapped to the mature miRNA sequences in the miRBase database (v22.1^69^). Additionally, the miRDeep2 software (v0.1.3^70^) was employed to identify putative novel miRNAs in mare endometrial cells.

A Principal Component Analysis (PCA) was performed (R package; v4.0.0^71^) to assess the overall similarity between examined groups. Next, DEmiRs (p_adjusted_ < 0.05, log2 fold change (log2FC) ≥ 1.0/log2FC ≤  − 1.0) were determined using R software (v4.0.0) using DESeq2 package (v1.28.1^72^). The unsupervised hierarchical clustering analysis present in the heatmap was performed using Euclidean metric distance calculated for examined samples.

To predict target genes for the identified DEmiRs, the GUUGle^73^, miRanda^74^, PITA^75^, rna22^76^ and RNAhybrid^77^ tools in the Tools4mirs server^78^ were used. The 5'UTR, CDS and 3'UTR sequences of horse protein-coding genes were used as potential targets. In addition, the binding free energy for potential miRNA-mRNA target pairs was calculated using the PITA, RNAhybrid, rna22 and miRanda tools. Only miRNA-mRNA pairs predicted by at least three of the five tools used and with a binding free energy below -10.0 kcal mol^−1^, were selected.

To explore the role of the revealed DEmiRs, the identified miRNA target genes were classified according to Gene Ontology (GO) and Kyoto Encyclopedia of Genes and Genomes (KEGG) categories to provide an overview of their biological functions and to assign them to specific cellular pathways and molecular mechanisms. Functional analysis of the identified target genes according to the GO database was performed using the clusterProfiler (v3.16.1^79^), DOSE (v3.14.0^80^), biomaRt (v2.44.4^81^) and AnnotationHub (v2.20.2^82^) packages of the R software, with the established criteria p_adjusted_ < 0.05. KEGG enrichment analysis was performed using clusterProfiler, DOSE and AnnotationHub packages of R software, with the established criteria: p_adjusted_ < 0.05.

Visual presentation of results was performed with R software using the ggplot2 (v3.3.5^71^) and pheatmap (v1.0.12^83^) packages.

### RNA extraction and RT-qPCR analysis

RT-qPCR was used to validate the NGS results and to determine the expression of genes that are potential targets of DEmiRs. The same tissue samples were used for both NGS and RT-qPCR. In detail, total RNA was extracted from endometrial tissue using a mirVana isolation kit (Invitrogen) according to the manufacturer's instructions. The concentration and quality of total RNA was determined spectrophotometrically. The A260/280 ratio was approximately 2. Total RNA was reverse transcribed using a TaqMan MicroRNA Reverse Transcription Kit (Invitrogen) with specific RT primers or a High-Capacity cDNA Reverse Transcription Kit (Applied Biosystems, Waltham, Massachusetts, USA) with RNaseOUT™ Recombinant Ribonuclease Inhibitor (Invitrogen) to examine changes in miRNA or mRNA expression profiles. The qPCR was performed using prepared cDNA, specific primers and probes, and TaqMan Universal PCR Master Mix II (Applied Biosystems). The qPCR conditions were set as recommended by the manufacturer: initial denaturation for 10 min at 95 °C, 45 cycles of denaturation for 15 s at 95 °C and primer annealing for 1 min at 60 °C. The qPCR data were analyzed as previously described^84^. Data are expressed as mean ± SD. Statistical analysis was performed using Student's t-test (GraphPad Prism software, version 7; GraphPad; San Diego, USA). Results were considered significantly different at p < 0.05.

To validate the NGS results, eca-miR-19a (Assay ID: 000395) and eca-miR-21 (Assay ID: 000397), miRNAs with a proven role during embryo implantation^37^, were selected for qRT-PCR. Reference miRNAs: RNU6B and miR-195, were selected based on our previous study^85^.

To determine the expression of potential DEmiR target genes, MUC1 ( Assay ID: Ec07038021_g1) and leukemia inhibitory factor (LIF; Assay ID: Ec07038654_m1), molecules that have been shown to play an important role during embryo implantation^19,51,59,60^, were selected. SDHA (Assay ID: Ec03470487_m1) and HPRT1 (Assay ID: Ec03470217_m1) were used as reference genes.

### Ethics approval and consent to participate

Procedures were reviewed and accepted by the Local Ethics Committee for Experiments on Animals in Olsztyn, Poland (Approval No. 51/2011). All methods were carried out in accordance with relevant guidelines and regulations. All methods are reported in accordance with ARRIVE guidelines for the reporting of animal experiments.

### Supplementary Information

Supplementary Information.

## Data Availability

The datasets used and/or analyzed during the current study are available from the corresponding author upon reasonable request. NGS results were deposited in the publicly available SRA (Sequence Read Archive) NCBI database under accession number PRJNA880660.
