# Supplementary material for: Bioinformatic analysis of endometrial miRNA expression profile at day 26–28 of pregnancy in the mare
Source: Sci Rep. 2024 Feb 16;14:3900. doi: 10.1038/s41598-024-53499-x (PMC10873421; doi:10.1038/s41598-024-53499-x)
Supplement: Supplementary file 1 — Supplementary Information. [file 41598_2024_53499_MOESM1_ESM.pdf]

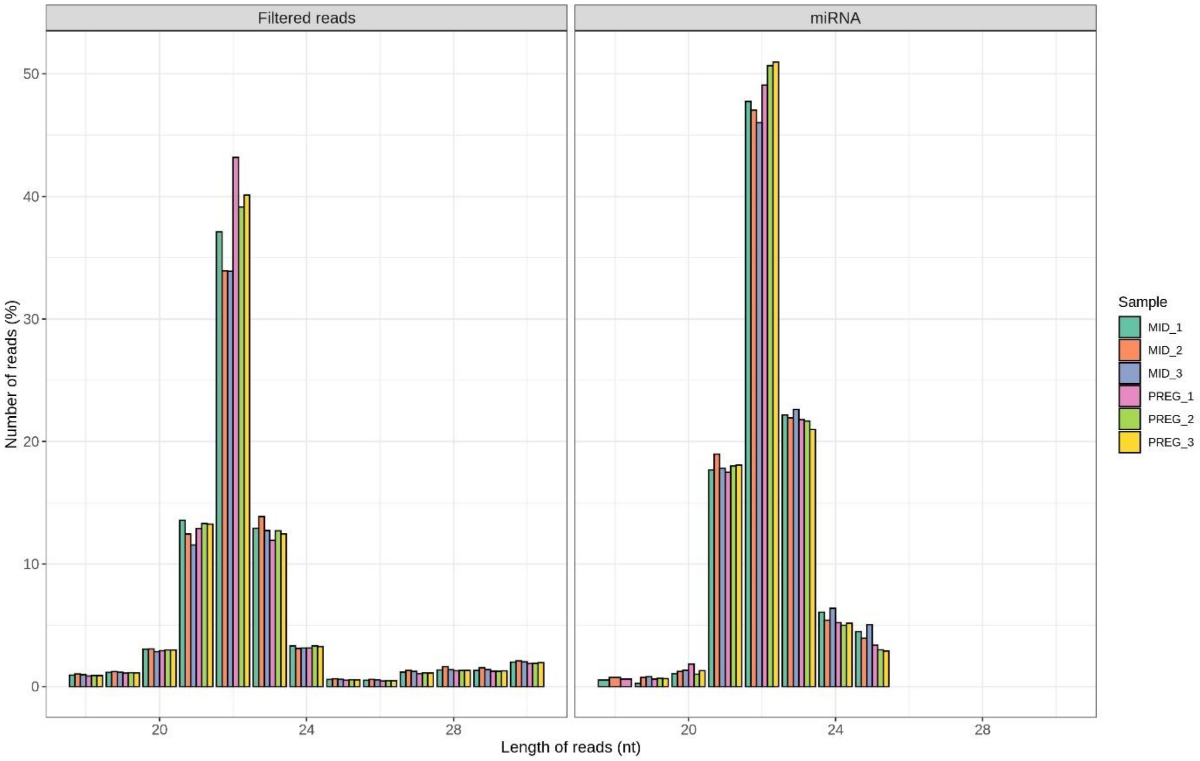

Supplementary Figure 1. The frequency of occurrence of different lengths of reads after quality control (small RNA) and miRNA sequences identified in mare endometrium tissue. MID: mare endometrium samples obtained during mid-luteal phase of estrus cycle (Day 10-12); PREG: mare endometrium samples obtained during pre-attachment period of pregnancy (Day 26-28); _1, _2, _3 - biological replicates.

Supplementary Table 1. The percentage contribution of ncRNA subtypes removed from particular samples during filtration process

No.	biotype	MID_1	MID_2	MID_3	PREG_1	PREG_2	PREG_3
1	Mt_rRNA	0.201	0.12	0.096	0.109	0.092	0.156
2	Mt_tRNA	0.231	0.234	0.236	0.188	0.322	0.141
3	Y_RNA	25.716	30.748	21.673	22.301	22.147	16.985
4	lncRNA	42.752	42.858	60.937	45.292	53.096	60.486
5	misc_RNA	0.123	0.068	0.071	0.149	0.104	0.102
6	rRNA	5.9	8.242	3.624	6.71	4.106	3.295
7	ribozyme	0.01	0.005	0.007	0.009	0.005	0.003
8	scaRNA	0.267	0.346	0.378	0.316	0.355	0.237
9	snRNA	0.353	0.192	0.139	0.306	0.227	0.2
10	snoRNA	24.168	16.898	12.568	24.402	19.35	18.158
11	vault_RNA	0.062	0.082	0.104	0.033	0.053	0.034

MID: mare endometrium samples obtained during mid-luteal phase of estrus cycle (Day 10-12);
PREG: mare endometrium samples obtained during pre-attachment period of pregnancy (Day 26-28);
_1, _2, _3: biological replicates

Supplementary Table 2. The length distribution of microRNA sequences

No.	Length (nt)	MID_1	MID_2	MID_3	PREG_1	PREG_2	PREG_3
1	18	2	3	0	2	0	0
2	19	1	3	3	2	2	2
3	20	4	5	5	6	3	4
4	21	67	77	67	57	54	56
5	22	181	191	173	160	152	158
6	23	84	89	85	71	65	65
7	24	23	22	24	17	15	16
8	25	17	16	19	11	9	9

MID: mare endometrium samples obtained during mid-luteal phase of estrus cycle (Day 10-12);
PREG: mare endometrium samples obtained during pre-attachment period of pregnancy (Day 26-28);
_1, _2, _3: biological replicates

Supplementary Table 3. Differentially expressed miRNAs ($p_{adjusted}$<0.05, log2FC≥1.0/log2FC≤−1.0) in mare endometrium samples obtained during pre-attachment period of pregnancy (Day 26-28)

No.	Identified	log2FC	$p_{adjusted}$
1	eca-miR-144	6.08772418214882	0.00902355459146846
2	eca-miR-32	3.04372149420267	9.15381986428184e-24
3	eca-miR-142-3p	2.83866860820362	1.17712312585935e-17
4	eca-miR-590-3p	2.74185336867513	4.51997193530313e-09
5	eca-miR-592	2.70988107166635	1.27026868311483e-07
6	eca-miR-19a	2.49806868289658	0.000158507180952522
7	eca-miR-21	2.19949826894319	7.31772331443689e-19
8	eca-miR-126-5p	2.15899987842839	1.05409686366122e-07
9	eca-miR-146b-5p	2.12962598326717	9.27048360926398e-08
10	eca-miR-101	2.06765408539917	7.62590480307866e-06
11	eca-miR-872	2.04057281732405	3.26236784122352e-06
12	eca-miR-218	1.99278560879973	5.24196129675564e-13
13	eca-miR-135a	1.98414728802564	5.24196129675564e-13
14	eca-miR-450b-5p	1.98015943381084	9.69990858206208e-11
15	eca-miR-450a	1.90735206744191	1.04438156843399e-06
16	eca-miR-148a	1.86978623874647	5.11949169344167e-11
17	eca-miR-7	1.79150155926176	2.02457883421319e-05
18	eca-miR-142-5p	1.67716902662137	0.00176479197072124
19	eca-miR-499-5p	1.66989353273355	1.92921606354902e-05
20	eca-miR-30e	1.66910057969774	1.1692289670901e-06
21	eca-miR-148b-5p	1.64772750562076	0.00905011878568218
22	eca-miR-628a	1.6338059463252	0.00933146266042318
23	eca-miR-140-5p	1.603146652496	3.32625628880221e-06
24	eca-miR-889	1.59480008472155	1.25375338860053e-12
25	eca-miR-542-3p	1.58680118721747	6.30298469743442e-06
26	eca-miR-450c	1.55291112388286	1.46823985499663e-05
27	eca-let-7f	1.55123521178443	5.27939479333486e-12
28	eca-miR-374a	1.54639038388089	5.24196129675564e-13
29	novel-eca-miR-13	1.51697401263316	1.04438156843399e-06
30	eca-miR-141	1.47618384928745	1.04438156843399e-06
31	novel-eca-miR-25	1.45072043268734	5.38853822132962e-05
32	eca-miR-369-3p	1.44620892887739	7.87219571255177e-05
33	eca-miR-136	1.43370619693344	0.000521243618810848
34	eca-miR-338-3p	1.41722861453279	0.0068491691161742
35	eca-miR-122	1.40584427330954	0.0132605663090793
36	eca-miR-98	1.37364020958252	7.71140080381362e-11
37	eca-miR-96	1.27888512129253	0.00103572479563183
38	eca-miR-429	1.24768614912355	0.000372498732031508
39	eca-let-7g	1.24763201541894	5.04014604863314e-10
40	eca-miR-451	1.23549947878994	0.00927672191385508
41	eca-miR-126-3p	1.22377240438664	1.25001167514328e-05
42	eca-miR-29b	1.19662554415688	0.0187604287159132
43	eca-miR-19b	1.17079847199336	0.000372498732031508
44	eca-miR-9a	1.14913119939594	0.0219973171090766

45	eca-miR-3959	1.1433623133214	0.00905011878568218
46	eca-miR-200a	1.13695732274945	0.00504079878733107
47	eca-miR-411	1.08296228112176	8.30953889717786e-06
48	novel-eca-miR-14	1.04346454051533	0.0319021804829817
49	eca-miR-191a	-1.03606760287213	9.18711738522022e-05
50	eca-miR-361-5p	-1.03662181955526	7.65442186562195e-05
51	eca-miR-744	-1.04455084095351	7.65442186562195e-05
52	eca-miR-423-3p	-1.05917139502394	1.19645317300489e-05
53	eca-miR-130a	-1.08991315664044	0.000874696831797852
54	eca-miR-504	-1.09731836762193	0.000606571231449232
55	eca-miR-30b	-1.10693138143317	0.0121289211275273
56	eca-miR-15b	-1.15322824513521	0.0121289211275273
57	eca-miR-129a-5p	-1.19648081717416	0.0441823568195229
58	eca-miR-193a-5p	-1.26639109375199	0.0132605663090793
59	eca-miR-150	-1.30737061431794	0.0116867090076397
60	eca-miR-652	-1.50543311846417	0.0162427020559935
61	eca-miR-500	-1.55017708130055	0.0125820481917822
62	eca-miR-129b-5p	-1.64616690777441	0.0162427020559935
63	eca-miR-491-5p	-1.66377561295988	0.0152162446398353
64	eca-miR-532-3p	-1.68161958294099	0.000374573284710516
65	eca-miR-214	-1.69354196101038	3.80640364099957e-06
66	eca-miR-1249	-1.83825024730339	0.00905011878568218
67	eca-miR-92b	-1.8465351635693	6.13966189915397e-05
68	eca-miR-193b	-1.87476952413008	0.00251083822396247
69	eca-miR-34b-3p	-2.06538801450415	1.47359640728318e-07
70	eca-miR-197	-2.11141914565513	7.46667314097204e-10
71	eca-miR-365	-2.19066866255095	0.000511180539816865
72	eca-miR-95	-2.27614336244148	0.000203772463779917
73	eca-miR-145	-2.3378721422323	1.37102095289166e-06
74	eca-miR-490-5p	-2.34378375870957	0.0354449332335071
75	eca-miR-129b-3p	-2.60660233864079	0.0228033798273244
76	eca-miR-331	-2.70888700764787	0.00122480679857449
77	eca-miR-1296	-3.48223175400516	0.00506147381039531
78	novel-eca-miR-49	-4.24415733196526	0.0462804918806293
79	novel-eca-miR-44	-4.75358121755298	0.0132605663090793
80	novel-eca-miR-42	-5.87463709615244	0.00100956967743194
81	novel-eca-miR-35	-7.99229110645569	3.66980940829777e-09

Supplementary Table 4. Targeted genes predicted to be regulated by differentially expressed miRNAs ($p_{adjusted}$<0.05, log2FC≥1.0/log2FC≤−1.0) identified in mare endometrium samples obtained during pre-attachment period of pregnancy (Day 26-28)

miRNA	TARGETED GENES
eca-let-7f	MCF2, NECAB1, DBX2, KBTBD3, IDH3A, ITGAM, VASH1, ZMAT4, ODF2L, MARVELD2, GLT6D1, ENSECAT00000039394.1, ENSECAT00000039404.2, PRUNE1, ENSECAT00000001706.2, CA14, OR2BB12, OR5P2, MAB21L2, TIFAB, BRS3, ENSECAT00000009402.2, CAPZB, RASSF7, ENSECAT00000010519.3, DEAF1, CRISP1, GALR2, ENSECAT00000015604.2, MBOAT7, VDR, YIPF3, ACER2, STOML2, YIPF4, MAL2, ENSECAT00000024914.3, ENSECAT00000024946.3, ENSECAT00000031256.2, ENSECAT00000036131.2, ADAMTS6, CEACAM19, SAMD10, OR7H3, SLC6A2, ENSECAT00000039175.2, ZNF512B, C2, TAF9B, ENSECAT00000049845.1, ENSECAT00000054574.1, ENSECAT00000066791.1, NASP, ENSECAT00000069568.1, ENSECAT00000075304.1, FBXL19, GNB5, TRIB3, ENSECAT00000002355.3, POMK, ENSECAT00000004300.2, ENSECAT00000005033.2, ABHD6, MFSD8, KCNRG, PIR, SLC10A3, NFATC3, PLXNB3, TRIM69, TMPRSS4, XKRX, ENSECAT00000020968.2, GCNT1, POU4F2, MLEC, ENSECAT00000024285.3, MPPE1, SLC11A2, ENSECAT00000038354.2, MFSD2B, ENSECAT00000048531.1, ENSECAT00000057286.1, ENSECAT00000060221.1, OR6C201, ENSECAT00000064290.2, ENSECAT00000068371.1, KDM5A, ASTE1, ADAM2, CEP85, RAP1GAP, FCHO2, ZMPSTE24, POSTN, ABHD11, MYH9, NDUFV2, LRWD1, TUBB, MAPK4, PDGFB, PTP4A3, AURKA, ENSECAT00000063944.2, KCNIP1, HLCS, HOOK1, BLM, MTMR14, ENSECAT00000076166.1, CHRND, HYOU1
eca-let-7g	MCF2, SORCS2, TSPAN13, PEX14, KBTBD3, FILIP1L, PHF21A, WDR5, ITGAM, OSMR, ODF2L, CD69, RMC1, ENSECAT00000033946.1, MARVELD2, DPP9, ENSECAT00000039394.1, ENSECAT00000039404.2, ENSECAT00000046835.1, FRMD4B, LHX4, PRUNE1, ENSECAT00000001178.2, ENSECAT00000001706.2, KLF17, OR2BB12, OR13C7, PCED1B, MAB21L2, POU2F2, ENSECAT00000007968.2, BRS3, PYGO1, ENSECAT00000010519.3, DEAF1, ADGRG3, CRISP1, VPS37C, LRRIQ3, MBOAT7, YIPF3, PPP1R7, ZGPAT, CLDN19, ENSECAT00000024946.3, MAZ, IKBIP, FAM166B, ENSECAT00000031001.1, ENSECAT00000031256.2, C23H9orf72, ENSECAT00000036131.2, CEACAM19, SAMD10, ENSECAT00000039175.2, ENSECAT00000040382.1, ZNF512B, C2, TAF9B, PXMP2, ENDOU, ENSECAT00000063912.1, GALP, PPP2R1B, ITPKC, ENSECAT00000069568.1, RNF215, ENSECAT00000075304.1, GNB5, GAD2, PRRG3, TRIB3, ENSECAT00000002355.3, POMK, NOBOX, OR4D2H, SLC44A1, ENSECAT00000004650.2, MMP28, ENSECAT00000005033.2, TULP3, CYP51A1, PDYN, ABHD6, ABHD12B, HACL1, TRIT1, TEAD4, TENT2, SLC10A3, EPN2, NFATC3, PDIA5, E2F4, PCED1A, CENPT, TMPRSS4, HEPACAM2, GCNT1, CSRNP2, MYPOP, NOCT, ENSECAT00000024285.3, CYRIB, ENSECAT00000038354.2, ENSECAT00000048531.1, ANGEL2, UIMC1, ENSECAT00000064290.2, ENSECAT00000068371.1, ENSECAT00000073136.1, KDM5A, CHRM3, RHOBTB1, TAF5L, RTP2, ACOX3, FBN1, MTUS1, POSTN, ABHD11, ADAM15, LRWD1, ATP7B, MAPK4, CACNA2D3, PDGFB, PTP4A3, HMGA2, SUN2, CNTN3, ENSECAT00000066102.1, DES, RNF165, HLCS, HOOK1, BLM

eca-miR-101	CROT, CLEC3A, OR10AM8, ENSECAT00000002680.2, OR10AM7, PHF21B, CHI3L1, ENSECAT00000071213.1, ARMCX5, GDPD1, EEF1A1, ENSECAT00000025249.2, ENSECAT00000039510.1, ENSECAT00000065685.1, ENSECAT00000067685.1, POLK, ENSECAT00000074104.1, ENSECAT00000077438.1, INPPL1, ENSECAT00000047518.1, ERCC6L2, RHOA, MDM1, ENSECAT00000065056.1, ZNF451, ENSECAT00000071702.1, MBP, ENSECAT00000073235.1
eca-miR-122	ICE2, CYP7A1, HSD3B2, MAB21L3, PAK5, GLRA1, HCRTR2, PCBP4, SLC6A9, LDHA, ADAM28, ENSECAT00000045235.2, FAM189A2, OR13P17, SESN1, OR6B9, OR5P1, OR6F1, TMEM251, RNF220, CD81, MIER3, AUP1, ZDHHC15, ENSECAT00000018985.2, HTR3A, IFT74, PAX9, IL6R, TCF7L2, B3GNT8, NR1H4, FOXP1, CEACAM19, MAST4, ILK, OR7G60, ENSECAT00000042147.1, APOC3, OR4D9I, ENSECAT00000052132.1, OR8T9, OR7G65, ENSECAT00000055241.1, ENSECAT00000056314.2, DENND1B, BPIFA2, GALC, TTC3, ENSECAT00000002806.2, KNOP1, OR10D4D, MARCHF3, ABHD3, TUBD1, LRR1, ENSECAT00000012921.3, ANXA4, GBE1, UBIAD1, RNF146, ELF1, GIT2, OR10H4X, PELI1, YPEL2, ENSECAT00000058812.2, CFAP107, TMPRSS13, SLC25A29, NAE1, FAM131B, MTMR11, WDR61, MED23, ENSECAT00000072717.1, ENSECAT00000074957.1, ZNF35, ENSECAT00000078291.1, GADD45B, USP6NL, EED, VAPB, ASIC2, EDNRA, ENSECAT00000045798.1, RICTOR, FNIP2, MRAS, ALPK2, ACSS1, JHY, HDAC9, PAXIP1, ALDOA, ENSECAT00000058546.2, KCTD1, ADSS2, ENSECAT00000061257.2, ENSECAT00000061988.1, MRPL22, ENSECAT00000063563.1, TRPM6, TPM4, SCAMP4, GDPD5, HIF3A
eca-miR-1249	PRF1, MAGI3, IDH3A, MPP2, LGB1, ST3GAL5, OXTR, LMAN1, CNOT3, SLC23A2, NDUFV3, TAF15, DTYMK, SLC17A5, ENSECAT00000019404.3, ENSECAT00000025818.3, CLCN7, PDGFB, PGGHG, ENSECAT00000071104.1, TNS2
eca-miR-126-5p	SMC2, ETV6
eca-miR-1296	FAM118B, NELFB, EIPR1, ADAMTS10, GAS7, NR1H2, LIG3, SORCS2, CYFIP2, RHEX, PPP1R16A, ENTPD2, RPS6KA1, TRIM35, FURIN, MYO18A, MAP3K9, MAPK8IP1, RNF44, UQCRQ, LRRC75A, S100A2, MUC20, ENSECAT00000011925.2, AK5, ENSECAT00000016569.2, CLN3, DBH, YPEL3, ALG12, B3GNT8, ENSECAT00000032798.2, ENSECAT00000034904.1, KLRG2, LYPD6, SULT4A1, ENSECAT00000062594.1, ENSECAT00000073930.1, ENSECAT00000076778.1, GSDMA, KIFC3, ASAH1, WDR45, ASIC2, ZFYVE27, C4orf48, TMEM101, RAB42, IRX4, IDUA, SDC3, ZNF496, ASH2L, ENSECAT00000041423.1, PDE6B, LYNX1, ANKRD65, RAPGEF5, ENSECAT00000072552.1, MMP28, C5AR2, ENSECAT00000007680.2, FBXO6, KRT75, HEXB, A1BG, MCIDAS, ANKRD13C, HNF1A, ENSECAT00000021728.3, ACP3, NFE2, TESC, FBXO21, NOP53, TYSND1, C1H1orf198, KREMEN2, EFNB1, KLK14, BAX, NOXRED1, ACTG1, BROX, SSC4D, CCT3, SOCS7, GGT6, ENSECAT00000033480.2, ENSECAT00000036618.2, LGALS3BP, ENSECAT00000044045.2, ATG7, MOCS1, SLC17A2, ENSECAT00000053846.1, ENSECAT00000061739.2, ENSECAT00000066772.1, SLCO2B1, ENSECAT00000068427.1, ENSECAT00000071840.1, ENSECAT00000073888.1, ENSECAT00000075243.1, CSF1R, ENSECAT00000076849.1, ENSECAT00000077659.1, ENSECAT00000077680.1, ENSECAT00000005372.3, CYTH4, GRHPR, CD22, ADAM12, CRYBG2, SIX5

eca-miR-129a-5p	GRM3, CCDC115, CYP4F124, PKD1L2, ANGPTL2, TCF21, UPK1B, CSDE1, ABCA9, AFG1L, PENK, CALD1, ENSECAT00000031945.2, DRB, ENSECAT00000039370.2, NT5DC1, SLC39A12, ENSECAT00000041944.2, ENSECAT00000043337.2, TRAV29DV5, ENSECAT00000043638.2, GLT8D2, LRRC30, LRRC15, ENSECAT00000006368.2, CYSLTR1, METTL24, MCOLN3, GEMIN2, TBP, TMEM9B, CCDC30, TFAP2D, SH2D6, RHAG, TRAF2, TNFRSF11B, CRBN, NCBP2, PNLIPRP1, PGPEP1L, ZNF404, ENSECAT00000020274.3, FAM162B, WEE1, NDUFA10, LMOD1, COQ8B, CTSS, ASMT, EXPH5, ENSECAT00000026628.2, ZNF18, MIPOL1, TNNT2, MEF2C, TRIM47, SERPINA11, GCAT, USP39, ENSECAT00000065626.1, ENSECAT00000069729.1, ENSECAT00000071703.1, ENSECAT00000074096.1, ADPRM, DYRK1A, DPRX, DDX46, CHRNB3, DDI2, ZADH2, ENSECAT00000004666.2, GJA9, ZNF80, ENSECAT00000005264.2, SPZ1, PPM1N, ENSECAT00000007680.2, ATL1, ENSECAT00000009451.3, RPL7, ARMCX5, RBM48, SMARCD2, CFAP20DC, ENSECAT00000014177.3, FAM3A, IFT46, TP53I3, BHMT, MEIKIN, CYP4F126P, NUCB2, AQP11, GAB2, DCUN1D3, TCP1, ENSECAT00000021731.2, AZI2, ENSECAT00000021789.2, EEF1A1, COPS3, WBP4, MMAA, TERF2, TADA2A, C13orf42, NECTIN3, CPNE3, ENSECAT00000061245.1, ENSECAT00000062422.2, POLR1G, ENSECAT00000066175.1, ENSECAT00000066180.1, ENSECAT00000066621.1, ENSECAT00000068382.1, TRDN, ENSECAT00000070826.1, ENSECAT00000072673.1, ENSECAT00000072789.1, ENSECAT00000072831.1, GSTO1, LSAMP, CYP8B1, ENSECAT00000037455.1, ENSECAT00000049673.2, ENSECAT00000055624.2, ENSECAT00000056949.2, ENSECAT00000001501.3, PPP4R3B, ANKFN1, CCL24, OSBPL10, IL36A, ENSECAT00000055011.1, IGF1, ASB1, ACO1, BCL11A, ENSECAT00000060069.2, EVI5L, MTX3, RSRP1, MSANTD2, PARVB, ZIC2, RCC1, TC2N, EIF5B
eca-miR-129b-3p	SYT17, PPM1H, ANXA9, EEF1A2, ENSECAT00000005264.2, EEF1A1, BGN, SELENOW, CEP85, ENSECAT00000061299.2
eca-miR-129b-5p	GRM3, CCDC115, CYP4F124, PKD1L2, ANGPTL2, TCF21, UPK1B, CSDE1, ABCA9, AFG1L, PENK, CALD1, ENSECAT00000031945.2, DRB, ENSECAT00000039370.2, NT5DC1, SLC39A12, ENSECAT00000041944.2, ENSECAT00000043337.2, TRAV29DV5, ENSECAT00000043638.2, GLT8D2, LRRC30, LRRC15, ENSECAT00000006368.2, CYSLTR1, METTL24, MCOLN3, GEMIN2, TBP, TMEM9B, CCDC30, TFAP2D, RHAG, TRAF2, TNFRSF11B, CRBN, NCBP2, PNLIPRP1, PGPEP1L, ZNF404, ENSECAT00000020274.3, FAM162B, WEE1, NDUFA10, LMOD1, COQ8B, CTSS, ASMT, EXPH5, ENSECAT00000026628.2, ZNF18, MIPOL1, SH2D6, TNNT2, MEF2C, TRIM47, SERPINA11, GCAT, USP39, ENSECAT00000065626.1, ENSECAT00000069729.1, ENSECAT00000071703.1, ENSECAT00000074096.1, ADPRM, DYRK1A, DPRX, DDX46, CHRNB3, DDI2, ZADH2, ENSECAT00000004666.2, GJA9, ZNF80, ENSECAT00000005264.2, SPZ1, PPM1N, ENSECAT00000007680.2, ATL1, ENSECAT00000009451.3, RPL7, ARMCX5, RBM48, SMARCD2, CFAP20DC, ENSECAT00000014177.3, FAM3A, IFT46, TP53I3, BHMT, MEIKIN, CYP4F126P, NUCB2, AQP11, GAB2, DCUN1D3, TCP1, ENSECAT00000021731.2, AZI2, ENSECAT00000021789.2, EEF1A1, COPS3, WBP4, MMAA, TERF2, TADA2A, C13orf42, NECTIN3, CPNE3, ENSECAT00000061245.1, ENSECAT00000062422.2, POLR1G, ENSECAT00000066175.1, ENSECAT00000066180.1, ENSECAT00000066621.1, ENSECAT00000068382.1, TRDN, ENSECAT00000070826.1, ENSECAT00000072673.1,

	ENSECAT00000072789.1, ENSECAT00000072831.1, GSTO1, LSAMP, CYP8B1, ENSECAT00000037455.1, ENSECAT00000049673.2, ENSECAT00000055624.2, ENSECAT00000056949.2, ENSECAT00000001501.3, PPP4R3B, ANKFN1, CCL24, OSBPL10, IL36A, ENSECAT00000055011.1, IGF1, ASB1, ACO1, BCL11A, ENSECAT00000060069.2, EVI5L, MTX3, RSRP1, MSANTD2, PARVB, ZIC2, RCC1, TC2N, EIF5B
eca-miR-130a	SMAD9, SLC17A9, NYAP2, ADGRL2, CEP68, SYNPR, TBP, SFRP2, ENSECAT00000033065.2, TRPC4, TMLHE, AZI2, RAD21L1, ENSECAT00000038237.1, ENSECAT00000057235.1, RASSF4, NUMA1, ENSECAT00000044564.2, CD83, HOMER1, FAM118B, SLC24A4
eca-miR-135a	EIF2AK2, ENSECAT00000021833.3, BMPR1A, OSBPL6, KLHL32, MACROH2A1, KIF22, LRRC73, GRP, ZMYND12, RFC3, ENSECAT00000045452.1, ENSECAT00000058670.1, ENSECAT00000061359.1, ENSECAT00000075187.1, ENSECAT00000007999.2, HNRNPDL, DNAJA1, ANKRD13C, CACNA2D4, GPAT3, SETDB2, ENSECAT00000033885.2, ENSECAT00000049396.2, DYM, ENSECAT00000045358.2, ARHGAP26, FOXN2, CD28
eca-miR-136	ENSECAT00000016780.2, STK31, ENSECAT00000024064.3, BPIFC, SLC2A9, CALD1, NHLRC3, SYNGAP1, ENSECAT00000052066.1, TESK1, ENSECAT00000004240.2, CCDC160, FANCC, KCNJ13, CYP2B6, ENSECAT00000009059.2, ENSECAT00000009251.2, EAPP, PBDC1, ENSECAT00000010519.3, CCDC30, ETFDH, SYCE3, PRPSAP1, SEC11C, KLF3, ENSECAT00000025941.2, RGS11, ANKRD45, FBXO9, ENSECAT00000036131.2, ARHGAP24, ENSECAT00000038670.2, PRPS2, ENSECAT00000039013.2, ENSECAT00000041527.1, ENSECAT00000042465.2, UBE2B, ENSECAT00000052701.2, ENSECAT00000055414.2, COX7B, ARPP21, MKKS, DIXDC1, ENSECAT00000067581.1, ENSECAT00000067887.1, ENSECAT00000068215.1, ENSECAT00000068639.1, ENSECAT00000069350.1, ENSECAT00000074810.1, ENSECAT00000076998.1, TEX33, NEUROD4, CRTAM, DYNLT1, INSC, RBM48, MXD1, VSNL1, ING5, ZNF23, MZF1, BAG1, TEX52, GPAT4, RBP7, ZC2HC1A, MSANTD3-TMEFF1, PTAR1, ENSECAT00000025249.2, ENSECAT00000039510.1, ENSECAT00000044991.1, ENSECAT00000051404.1, TGFBR1, REELD1, ENSECAT00000057625.2, MMAB, POLR1G, AKAP7, AP2M1, CAMK2G, ZNF662, RBM34, ENSECAT00000004560.3, ACAP2, VWA5B1, CDC42BPB, SLC25A37, PTCHD4, CCDC91, MEAF6, DLD, STAT5A, RNASE4, TMEM52B
eca-miR-140-5p	FNIP2, POLR2B, HUWE1, DSC3, CDH19, KLHL8, RBMS2, ADGRB1, ENSECAT00000003103.2, CACYBP, RRAGA, ENSECAT00000008826.3, PYGO1, GATAD2A, AK5, ENSECAT00000014890.2, ORC4, MMD, ZNF226, HSCB, AMDHD1, ENSECAT00000043573.1, ENSECAT00000043709.1, DENND5A, ENSECAT00000051664.1, ENSECAT00000052483.1, ENSECAT00000053729.1, ENSECAT00000063397.1, ENSECAT00000064869.1, ENSECAT00000001505.2, ENSECAT00000009784.2, APOH, CERKL, SMAP2, PLEK, ENSECAT00000041615.1, ENSECAT00000054712.1, KCNK3, ENSECAT00000065905.1, ENSECAT00000070237.1, TBC1D31, ENSECAT00000077516.1, DISC1, NPFFR2, HEATR6, CILK1, SSR1, TRPM1, NUDCD1, ENSECAT00000054861.1, EPC2, ENSECAT00000058685.1, CSF3R, TBC1D5, TXNDC8, SLC4A8, ROBO1, TMEM52B
eca-miR-141	C1QTNF3, PDE6B, PIGK, UBASH3A, OTOR, IQCM, ENSECAT00000067749.1, OR2L27, ASPN, BARX1, GSTO1,

	ENSECAT00000057037.1, EMX2, MAST3, ASB1, NUCKS1, LEP, CARNS1, ABCC6
eca-miR-142-3p	ENSECAT00000013718.3, CEP128, COQ9, STX6, OR10D5H, ANGPTL3, TM2D2, TMLHE, HRH2, YES1, VPS26A, PIK3R6, PTK7, GRHPR, SCN8A, UBE2D1
eca-miR-142-5p	MRLN, ASB9, RHOA, BUD13
eca-miR-144	AVEN, TOGARAM2, NT5C2, ISM1, ENSECAT00000011874.3, SNRPB2, STKLD1, GPR65, ODF1, OR6A2, ENSECAT00000011251.3, PRCP, AFTPH, ARL6IP5
eca-miR-145	ENSECAT00000008028.3, ENSECAT00000009232.3, CFAP73, ENSECAT00000011900.2, ENSECAT00000017763.3, ENSECAT00000022011.3, SEPTIN9, LARP4, NRP2, ENSECAT00000049576.2, ENSECAT00000000570.2, FOXO1, ENSECAT00000004124.2, ENSECAT00000006631.2, ENSECAT00000006754.2, FAM81B, PNLIP, TMOD2, BRMS1L, MGAT1, HIRIP3, ENSECAT00000019327.2, UCHL1, FAM162B, UBQLN1, SVBP, A4GALT, TMCO4, CNDP1, SNAP25, KRTCAP3, MLPH, ARSK, SAP30BP, MAD2L1BP, GPR25, JADE2, FUBP1, ERBIN, TPM3, ENSECAT00000044878.1, ENSECAT00000045041.2, ARPP21, ENSECAT00000050667.1, ENSECAT00000055448.1, GNE, NNMT, ENSECAT00000000272.3, DDX6, ENSECAT00000002120.2, S1PR3, SLC27A6, RNF115, EPSTI1, TRIM45, CHMP2B, TBCEL, CARNMT1, DPAGT1, ENC1, SDCBP2, TMEM184C, HNF1A, RBBP7, HAS2, WIPI2, ARSG, STRADA, ARMCX2, ENSECAT00000033049.2, SETDB2, WDR3, ENSECAT00000034819.1, LINGO3, ENSECAT00000046588.1, FAM219A, SHOC2, EXOSC3, ENSECAT00000068493.1, RGS9, LIN54, ADD2, MRO, TRIM10, ARHGEF7, HAP1, OPN3, ENSECAT00000048812.2, ENSECAT00000052960.1, EIF4A3, MORC1, CWF19L2, ANKS6, CFTR, PCNX4, PLEKHB2, HBS1L, PLCL2, CARS2
eca-miR-146b-5p	TYRP1, SMIM19, NT5DC1, TMEM140, GPC1, EIF2B3, UGT1A1, PIGX, ENSECAT00000035510.1, MIPOL1, ENSECAT00000058728.1, SHC4, LAPTM4A, MGAT4D, NOX1, APOH, OR2T1B, ENSECAT00000041282.1, GIMAP8, ENSECAT00000061358.1, ABCC3, SSH1, ARHGAP4, CDHR3, LMBR1, MARCHF5, ENSECAT00000066575.1, ENSECAT00000067211.1, ENSECAT00000068160.1, KAT7, IFT22
eca-miR-148a	MMP20, AAMP, GLOD5, SYNE1, LRP2, TMEM52B, SLC19A1, MBTPS1, PDHA1, RNF220, FAM114A1, TMEM150A, OR5P3B, SLC15A3, ENSECAT00000059768.1, CYP51A1, POLE2, TAF7L, SRFBP1, SHMT1, ENSECAT00000021975.2, ANKRA2, TIA1, ENSECAT00000024888.3, CAP1, ENSECAT00000032602.2, ENSECAT00000046205.2, ENSECAT00000061180.1, ENSECAT00000061430.2, ENSECAT00000072667.1, ENSECAT00000073537.1, ENSECAT00000075706.1, ENSECAT00000035172.1, DNAJC10, CRYBG3, AGRN, WDR47, ERICH3, PTGES3, ENSECAT00000070759.1, GPSM1, ROBO1
eca-miR-148b-5p	ACAP1, ZNF615, MEMO1, NECTIN2, CSRNP2, BROX, ALPK2, ZMPSTE24, IL13RA2, ENSECAT00000056062.1, ACVRL1
eca-miR-150	TBC1D8B, CDH19, SORBS2, SAP25, ENSECAT00000009906.2, MELK, COQ8B, PRNP, DIXDC1, SARDH, HCLS1, KRT4, NIM1K, CHMP4B, TMEM26, LY6D, ENSECAT00000006781.3, CCDC136, LRCH4

eca-miR-15b	SPATS1, PRKD3, AASDH, PLPPR2, GDAP1, TNFRSF8, FGF10, EIF4EBP2, MYO1F, VARS2, CALML4, ENSECAT00000023502.3, SHH, MVP, PPARGC1A, FAM189A2, EPM2AIP1, RBM47, LDOC1, OR10AD1, OGFOD1, MHCB3, ZACN, NECTIN2, DRD4, A1CF, RAB17, RETSAT, TBC1D22A, LMBR1L, DRD2, GTDC1, FAM43A, ENSECAT00000015736.2, YIPF3, TOR4A, RBMS3, MKRN1, RGMB, ENSECAT00000019472.3, CDKN2A, ENSECAT00000019643.2, NLRP10, GPR37L1, SFTPB, TSPEAR, AURKC, TEDC1, PLAUR, RHBDD3, ENSECAT00000024914.3, SNX8, ENSECAT00000026015.2, ENSECAT00000026055.2, KRTCAP3, POLR3G, TSPAN32, UBE2S, ENSECAT00000031007.1, ENSECAT00000032732.1, SLC12A8, ENSECAT00000036222.1, CAPZB, ENSECAT00000040034.2, IGF1, ENSECAT00000044783.2, FADS1, ENSECAT00000049453.2, OR6Z10, ENSECAT00000052102.2, ENSECAT00000052621.1, NPDC1, GTF2H4, ENSECAT00000063999.2, GAS1, TMEM250, TARS2, XBP1, ENSECAT00000067822.1, NACC2, SARDH, LPAR4, COL23A1, SPTBN4, ENSECAT00000076778.1, ENSECAT00000077323.1, ENSECAT00000077955.1, OXSM, OR9R10, OR9R9, KRT72, DNASE1L1, NECTIN3, CYP51A1, CACNG4, TTC36, CYP46A1, ENSECAT00000008276.2, PI4K2B, CCNY, EGR4, HSPA8, TMEM241, ELL3, LGI2, CPEB4, BTBD10, SLC35F6, KRT4, TRIB1, SPTLC1, GDF7, MLEC, TKTL1, BCAP31, BPIFB2, E2F1, CCL5, ENSECAT00000037179.2, EFNA2, CCNJL, ENOX1, GAP43, WDR25, ENSECAT00000049678.1, PABPC4, NRSN2, GPAT4, C22H20orf144, SMARCD2, PALM, TMEM65, ENSECAT00000064055.1, AMH, METTL4, ENSECAT00000070916.1, ASB9, FAM107A, ENHO, ENSECAT00000077630.1, MTSS1, H2AC19, MCU, NUDT13, ENSECAT00000023525.2, ALG3, ENSECAT00000042385.1, SLC18A3, THAP1, ENSECAT00000003845.3, CTTN, NUMA1, TTLL7, SNX16, USP40, TFAP2A, DCLK1, CMTR1, MON1B, TRIM37, CD22, ADAMDEC1, PDE3B, SLC4A7, LALBA, TMEM235, ZC4H2, ATP5MC1, NKD2, ENSECAT00000066094.1, C25H9orf78, VIP, SF1, CHRNA1
eca-miR-191a	CTSO, GDI1
eca-miR-193a-5p	UPK2, DENND3, PPP6R1, ENTPD2, CD38, LCN2, DZIP1, HMGXB4, KCNJ5, HMX3, KDM2A, MB, ENSECAT00000036781.1, ENSECAT00000041266.2, ANKRD11, CACTIN, CCDC102B, BEAN1, ENSECAT00000051606.1, ENSECAT00000052111.1, ENSECAT00000053245.2, ENSECAT00000000513.2, CLEC14A, TSEN54, ILK, ENSECAT00000008394.2, RPL7A, PIP5K1A, ENSECAT00000009807.2, PLA2G2A, ZPR1, PNPLA2, SERPINA3, UTS2B, WFIKKN2, RPS8, ENSECAT00000017301.3, N4BP2L2, ENSECAT00000017652.3, TNFRSF21, ERF, TGFBI, ELMOD3, CTSC, ELL2, ADCK2, TEX35, TRIM25, FAM234A, SERPINA9, ZNF580, ENSECAT00000033982.1, KIAA1671, TRIM16, OR8S15, ENSECAT00000037996.2, ENSECAT00000039814.1, OR8S35, GNLY, ENSECAT00000050376.2, ENSECAT00000050843.2, CDC25B, ENSECAT00000055589.2, SHTN1, SERPINA11, ENSECAT00000060886.2, ENSECAT00000063617.2, ENSECAT00000065811.1, PCSK7, LEO1, MINPP1, ENSECAT00000069231.1, GLDN, ENSECAT00000073592.1, ENSECAT00000073972.1, ENSECAT00000076139.1, SLC35E3, PLCB1, ENSECAT00000078711.1, KPNA7, TCF3, SLC25A53, UBAP1, NECTIN3, PDYN, FBXW8, SLFNL1, SLC39A3, LRRC38, MCCC2, CD300LB, FAM71A, TARBP2, WLS, TMEM38B, HNF1B, C21H5orf51, PRR16, PIAS3, SLC28A2, OPN1LW, HELQ, MMP16, SIGMAR1, CAT, CCDC8, HS1BP3, WARS2, LDHC, GABRB3, EIF3D, ENSECAT00000024707.2, GZMA, AQP2, ZNF385A, CNBD1, PRUNE2, PLEK, C19orf25, C6H12orf4, ENSECAT00000049678.1, TMEM176A, MECP2, SLC22A1, ENSECAT00000063021.1, SLC66A2, GAP43, LPO, ENSECAT00000077497.1, ENSECAT00000077630.1, ASCC1,

	RGR, FOXL1, SLC6A1, SEC16A, RXRA, PHF8, ENSECAT00000045353.2, MGRN1, ASIC2, HFE, TGFB3, SS18, UST, SEPTIN9, FZR1, NACC1, BFAR, SEC22B, MLNR, KCNQ5, PAAF1, MXRA8, GPR83, CARNS1, ENSECAT00000071781.1, RNF150, OPHN1, DIO1, TMEM130, CCT6A
eca-miR-193b	EGR2, ENSECAT00000011985.3, TNFRSF8, ACTN3, FCHO2, TRIM38, SEPTIN1, ENSECAT00000026786.3, PLA2G7, TPPP3, ANTXR1, FLVCR1, MARVELD3, ENOPH1, PWP1, ASNSD1, ENSECAT00000054127.1, RDH10, VCAN, ENSECAT00000049495.1, SLC5A10, DPP9, ASDURF, ELMO2
eca-miR-197	CROT, ENSECAT00000018540.3, ENSECAT00000024621.3, SLC6A9, ILT11B, VPS26C, LAIR1, ENSECAT00000059866.2, ENSECAT00000067750.1, ENSECAT00000072614.1, CHRNA6, OR4D2H, ITPRIP, KRT76, KRT3, RAD51D, PXN, ZC3H15, EHD2, PRUNE2, PDIA5, TMEM160, C1H10orf53, ENSECAT00000059926.2, ART5
eca-miR-19a	STAR, MTM1, GM2A, MLLT6, ENSECAT00000043917.2, ENSECAT00000056128.2, RASA1, HLF, MARCHF7
eca-miR-19b	STAR, MLLT6, REXO2, AFMID, GABPB1, NPRL3, ENSECAT00000060886.2, FMO5, KLHL6, ENSECAT00000043917.2, ENSECAT00000056128.2, MARCHF7
eca-miR-200a	PKP2, DBF4, NUBPL, BPIFB4, PDE6B, EIF4E3, UBASH3A, TMPRSS2, WDR4, ENSECAT00000067749.1, RCOR3, ZNF551, GGNBP2, RBBP7, ENSECAT00000066772.1, MAST3, ATG16L1, ASB1, KNDC1, ENSECAT00000072018.1, ENSECAT00000073427.1
eca-miR-21	DERPC, CACYBP, NFIB, RXYLT1, SKP2, ENSECAT00000064504.2, IRAG1, VAMP7
eca-miR-214	ENSECAT00000009796.3, MYO5A, ODF2, DHRS2, ATP2A3, EPHA4, MIB2, CYP4F124, LRTM2, PEG3, RPS6KA1, GTPBP1, ESPN, OSBPL6, LCN2, IL1F10, FGF6, PLA2G3, ARAP2, KLHL42, ENSECAT00000045547.2, SAMD4A, RBFOX3, BBX, ENSECAT00000000493.2, RSPH9, CXCR4, SCPEP1, STS, CCDC97, CRISP1, B9D2, ENSECAT00000013513.2, SLC22A13, SLC22A10, ENSECAT00000016573.3, MIGA1, HBZ, CXXC5, CD180, CREB3L4, PRR35, DIPK1C, GNA12, ENSECAT00000022069.2, SLC39A1, PTTG1IP, RHBDD3, FAAH, SLC13A4, MLPH, TMEM53, EQUCABV1R901, CCDC184, CAPN10, ENSECAT00000043551.1, CACNG6, XPR1, ENSECAT00000045983.1, SLC29A1, ENSECAT00000046655.2, ENSECAT00000048427.2, ENSECAT00000053613.1, ENSECAT00000053870.2, RAD54L, AGPAT1, ENSECAT00000055327.1, ENSECAT00000056037.2, GPBP1, ENSECAT00000058171.1, SOX3, NUP85, KIAA0930, ENSECAT00000060095.1, SLC9A6, ENSECAT00000063422.1, ENSECAT00000065130.2, ENSECAT00000065442.2, ENSECAT00000066497.1, ENSECAT00000067596.1, LBHD1, TRPC4, ENSECAT00000076438.1, ENSECAT00000078041.1, R3HDM4, TMEM35A, OR6X1, SLC25A29, NMRK1, ERGIC1, CERS5, ENSECAT00000008003.2, ODR4, ENSECAT00000009115.3, THY1, ENSECAT00000010690.3, GRPR, PCED1A, CPEB4, AQP11, KPNA1, HSF5, PREB, WNT11, MYPOP, GPRC5B, EIF3D, DRAXIN, PTGIR, ABCE1, HRH2, CSMD2, ZNF697, DTNB, ENSECAT00000046282.2, ENSECAT00000046735.2, ENSECAT00000053342.1, PDIA4, MIIP, ENSECAT00000065853.1, JAML, ENSECAT00000066124.1, ENSECAT00000066958.1, ENSECAT00000069288.1, ENSECAT00000070442.1, ENSECAT00000073831.1, ENSECAT00000076844.1, ENSECAT00000078012.1, ENSECAT00000078783.1, DUSP13, CYP8B1, ENSECAT00000003700.2, RASSF4, SLC29A3, PSEN1, LY6D, FAM76B, STX3, PLEKHG3, TSNARE1, NBN, SH2D2A, XAB2, SEC24C, SEPTIN11, TAB3, SLC5A10, HGS, FGF4, PIEZO1, ENSECAT00000056010.2, RAP1GAP2, GAB2, SRRM1, CPA4, NKD2, SMG5, CSMD3, RAB11FIP1

eca-miR-218	RNF44, MYLK2, TRPM8, APOE, MDM2, MAB21L4, RNF157, RABGAP1L, C26H21orf91, GSTO1, SPRYD3, CORO1C, CCDC81, ENSECAT00000043601.2, CXCL12, CPNE8, TPK1, LCP2, DHX29, TXNL1, CTSZ, CPSF7, CLTC, DPEP2, PAFAH2
eca-miR-29b	OTOGL, SCARA5, ACKR3, SIRT3, ENSECAT00000006710.2, AKR1D1, AMIGO2, ENSECAT00000025934.2, ENSECAT00000035526.1, CHRM2, TCP11L2, PSKH1, GGA2, IDO2, FBXO33, RARS1, ENSECAT00000031372.1, TMEM121B, ENSECAT00000046342.1, ENSECAT00000049981.1, ENSECAT00000052056.1, ENSECAT00000053826.1, ENSECAT00000054714.1, ENSECAT00000063593.1, NOTCH2
eca-miR-30b	TBCE, SENP1, GXYLT1, ENSECAT00000072339.1, ME3, FAIM2, RCBTB2
eca-miR-30e	CCDC126, SERPINB12, C23H9orf64, ENSECAT00000057723.1, ENSECAT00000060709.1, RBM46, ENSECAT00000078508.1, ENSECAT00000006049.2, APOH, TTC17, ENSECAT00000035453.2, ENSECAT00000047672.2, ENSECAT00000048531.1, ADIPOR2, ENSECAT00000056394.2, ENSECAT00000068784.1, ENSECAT00000075450.1, PSAP, SEL1L3, ATG5, NCOA1, GYS1, CEP57
eca-miR-32	NCR2, B4GALT7, TWF1, PDE4D, NLRP5, ZNF671, NKPD1, FAM189B, ENSECAT00000070759.1
eca-miR-331	ACVR1B, PLIN1, AP1S3, MMACHC, FGFR3, TNNT2, ENSECAT00000024488.3, TRPM2, SMTNL2, ZNF444, LRRC28, CCN6, ZNF133, UBE2Q1, HIPK4, RPL28, ENSECAT00000062741.2, NR2C2AP, NOBOX, OR2A7, EREG, SLFNL1, ZNF169, MFSD14B, EVL, REPIN1, SHF, ISX, OR2T1B, VAPA, HMMR, ENSECAT00000063860.2, VSTM4, TRAM1L1, GUCD1, WDR81, MOBP, MDFI, ZNF692, SLC4A8, ABL2, PML, DSG2, ENSECAT00000077053.1, MORC4
eca-miR-338-3p	CRYGB, KRT6C, ENSECAT00000022011.3, ENSECAT00000023767.3, NSD3, ENSECAT00000049576.2, PRXL2C, RDH8, PHKG2, KPRP, SBK1, PAPSS2, NUDT1, SPAST, ENSECAT00000011551.2, ALDH4A1, SPNS1, ERI3, GAL3ST1, GSK3A, AMIGO2, ENSECAT00000022254.2, TRHR, TDRD10, STK19, EEF1A2, FBXW7, LGMN, ZDHHC15, NFIB, RMND5B, ENSECAT00000003030.3, OR13F8, TRAPPC4, ANKRD33, NMNAT1, CA4, ENSECAT00000038771.1, OR13D2H, GZMM, ENSECAT00000060936.1, SCN2B, PTPN20, RSPO4, ATG9A, SRCIN1, ACOX3, AGL
eca-miR-34b-3p	IL12RB2, ENSECAT00000005327.2, RALGPS2, KCNJ16, DDX52, E2F1
eca-miR-361-5p	BBX, IKZF2, RNF13, CHGB, OR8S8, OR8T9, GTF2H4, FBXL19, ENSECAT00000078030.1, JAML, OR10D5H, ENSECAT00000004781.2, BMP5, ATL1, CALCRL, HSPA8, PRADC1, UTP15, EBPL, MKS1, MYO3A, WSB2, ATRAID, STAU1, LEO1, ZNF630, AGO2
eca-miR-365	ENKUR, TRIM52, TRIM41, ENSECAT00000048588.1, ASPN, EOGT, PCDHGA4
eca-miR-369-3p	NLRC4, PTAR1
eca-miR-3959	PLAT, GALNT12, HPCAL4, ENSECAT00000020491.2, MAP2K3, BTBD3, ENSECAT00000043298.1, GNB1, TRIM37
eca-miR-411	HDAC2, LYSMD4, OR7E224, ENSECAT00000023032.3, UCHL1, ENSECAT00000075786.1, PRRG3, TMEM82, HSPA13, OIT3, PTPN20, ZFAT, RUNDC1
eca-miR-423-3p	MTM1, PARD3, HCRTR2, CACNB1, ALKAL1, PLA2G6, CACNG8, CA14, CEL, SLC23A1, NKX6-1, IRX6, B4GALT2, ILKAP, KLF13, TMEM200C, BACE2, CDKN1C, HECTD3, TMEM216, DDI2, PDXP, ME3, TP53INP2, EPN2, ATP6V0D2, D2HGDH, ENSECAT00000019770.3, GEMIN8, SYNPR, NTAN1, RASSF4, HTRA3, CHP2, SPSB1, C2CD4C, MLLT1, SERINC2
eca-miR-429	GABRG1, NUBPL, PDE6B, UBE2E3, EFHC1, CACNG7, PIGK, TYR, ENSECAT00000017652.3, CNOT6, CCDC38, IRAK3,

	ENSECAT00000066951.1, ENSECAT00000018712.3, ALG8, C2H4orf33, SGPL1, MARCHF8, LAMA1, S100PBP, JKAMP, HIPK3, MIP-2BETA, FRMPD2
eca-miR-450a	ST3GAL6, TRAV29DV5, USP39, ENSECAT00000021197.2, C2H1orf50, ENSECAT00000038822.1, MACROD2, FLT4, ENSECAT00000071275.1, ANKRD34B, POC5, PSD3, TACC1, RTKN2, KIFBP, USP6NL, MCOLN1
eca-miR-450b-5p	RERG, TECRL, LIN28B, NT5DC1, NFATC2, SCO2, NAT9, ZNF226, MACROD2, FAN1, MTMR10, GPC3, ENSECAT00000072908.1, C26H21orf91, NR1D2, ENSECAT00000019770.3, VRK1, ARSG, SLC12A1, PSD3, RTKN2, KIFBP, ENSECAT00000045279.2, GRM2, CAST, ENSECAT00000067263.1, TDRD1, ENSECAT00000076355.1
eca-miR-450c	ST3GAL6, TRAV29DV5, USP39, ENSECAT00000021197.2, C2H1orf50, ENSECAT00000038822.1, MACROD2, FLT4, ENSECAT00000071275.1, ANKRD34B, POC5, KCNK2, PSD3, TACC1, RTKN2, KIFBP, USP6NL, MCOLN1
eca-miR-451	ENSECAT00000000855.2, ENSECAT00000062658.1, TMEM214, ENSECAT00000068086.1, ENSECAT00000069381.1, ENSECAT00000073007.1, ENSECAT00000078772.1
eca-miR-490-5p	KIZ, ENSECAT00000046644.2, TAS2R3, SLC6A9, ENSECAT00000007991.2, LRRC19, UBQLN1, C2, WDR61, ENO1, G6PD, PLP1, UBASH3B, DYNC1LI2, POLR3C, PDLIM4, ENSECAT00000036182.1, ENSECAT00000039548.1, ENSECAT00000049856.2, ZCCHC4, ENSECAT00000042454.1, ENSECAT00000059260.2, FAM217A, MYCBP2
eca-miR-491-5p	FGG, CLSPN, HUWE1, FOXP3, PABPC1L, TCERG1L, PHYHIP, MB, SLCO1C1, ENSECAT00000043093.2, ENSECAT00000051849.1, OR6B2C, FOXO1, ENSECAT00000002680.2, APOA4, FANCC, HCCS, LPCAT2, SLC37A3, RETSAT, XRCC1, HPX, PCYT1A, ENSECAT00000031218.2, ZNF575, SAMD10, OR8S25, STK33, ENSECAT00000056557.1, GRK6, NECTIN2, ADAMTS6, MYOD1, KCNC1, NTAN1, WSB2, CXCL11, SPATA21, BCL2L1, APOBEC4, PCBP2, RALY, ZNF185, ENSECAT00000074432.1, FSCN1, C1QC, FHOD3, PLBD2, CUX1, ENSECAT00000050210.2, ITK, SPARC, ENSECAT00000051542.2, ENSECAT00000052218.2, MTMR6, PAPPA, ENSECAT00000060107.2, RHBDD2, PBX1, REEP6, ENSECAT00000064249.2, GTPBP8, TRIM66, ZC4H2, RBCK1, HERPUD1, RAPGEF3
eca-miR-499-5p	FGF10, DMRT3, FRMPD1, SLC30A8, FAM214B, ANXA13, PTP4A1, GGNBP2, PTPRR, CYP4F126P, ENSECAT00000077316.1, GARRE1, LSM14A, CAVIN1, LDLRAD3
eca-miR-500	PLAG1, ENSECAT00000015056.3, ENSECAT00000017839.3, ADGRL3, TMEM52B, TAOK1, ZFP2, ENSECAT00000006580.3, SHTN1, ZNF304, NECTIN2, FGB, MCRS1, ZNF134, BHLHE40, LYZ, DNASE2B, NFKBIA, C1orf68, CNTN1, SARDH, CYP46A1, PON3, CFAP20DC, CDKL2, RRAS2, RAD21L1, KCNS3, WARS1, POLR1G, DLEU7, ENSECAT00000003684.2, WDR72, LRRIQ1, CDV3, AK4, PLPBP, AMOTL2, MYB, PDE3A, ENSECAT00000061299.2, CPA4, GORASP1, MSH3
eca-miR-504	CSNK1D, GPIHBP1, ROM1, ACAP1, TBK1, LZTS3, SYT12, ENSECAT00000030845.1, ENSECAT00000031956.1, CPA5, CD14, MYLK4, ENSECAT00000040021.2, ENSECAT00000040120.2, ACAN, ENSECAT00000041184.1, ENSECAT00000042693.2, PLCH1, ENSECAT00000045114.1, ENSECAT00000048128.2, ENSECAT00000048267.2, SGK1, GOLM1, PIN1, ANKRD55, BSCL2, RIC3, ZBTB3, RHBDD3, NDOR1, ENSECAT00000026777.2, ENSECAT00000038087.2, PLRG1, ENSECAT00000043551.1, FAM172A, TCTE1, ENSECAT00000049850.1, STK33, ALK, ENSECAT00000000711.2,

	CKAP4, GH1, TMEM30A, CCKAR, GDI1, TMEM150C, DUS2, CYFIP1, MARCHF3, SLC16A8, ASIC3, SELENOW, SMIM20, TMEM72, KLHL40, PRDM2, KCNC4, ATXN7, GRM2, NKPD1, ABHD2, DPP9, ENSECAT00000056010.2, ANHX, MLLT1, ARHGEF2, ACAD8, MEN1, TBC1D16, NDUFA9, ENSECAT00000075173.1, ENSECAT00000078391.1
eca-miR-532-3p	ZIC3, IGFBP5, TNFRSF8, TFF2, KCNV2, MYL10, SFRP5, NHLRC3, STAG2, ZDHHC2, RBBP9, TNNI3, TSPAN31, ENSECAT00000046722.2, OXSM, PNMA3, HS3ST2, DUSP9, CCDC81, CCNK, HDC, TMEM39A, SF3B4, HINFP, ENSECAT00000044119.2, GIMAP7, ENSECAT00000072717.1, CABP1, GADD45B, NNT, FBXO31, UGGT1, NPTN, TMEM235, SYMPK, MINDY4B, RAPGEF3, CCDC174
eca-miR-542-3p	FKTN, CRYGA, MORC1, SNX20, IL31RA, RGN, COLQ, CD244, R3HCC1, HPS5, MC4R, KIF2B, BDH1, F12, MYBPH, WASF1, TLCD3B, ABLIM1, GFRAL, ENSECAT00000030505.2, ITGAL, ENSECAT00000037097.1, EGLN2, ENSECAT00000061620.2, NACC2, ENSECAT00000070262.1, RIMBP2, ERGIC1, DEPDC1, NEPRO, MAP2K6, SNX30, GLRA4, FBXO32, BRCC3, ACP6, CD58, ENSECAT00000036076.2, EOGT, ENSECAT00000059849.2, ENSECAT00000062349.2, ENSECAT00000068163.1, ENSECAT00000071040.1, ENSECAT00000073757.1, ANAPC16, ACHE, HCFC2, LZTR1, LPIN3, TMEM43, USP35, ENSECAT00000048077.1, COL4A6, ENSECAT00000058601.1, ZNF335, ENSECAT00000062835.2, CREM, PLEKHS1, ENSECAT00000075653.1
eca-miR-592	ENSECAT00000012247.2, ENSECAT00000012279.2, IL12RB2, SS18L1, PDXDC1, BNC2, ENSECAT00000006329.2, ENSECAT00000032649.1, NR1D2, ENSECAT00000021760.2, ASB9, ENSECAT00000063021.1, VPS35L, ENSECAT00000006831.3, ENSECAT00000006918.3, ENSECAT00000006979.3, ASCC3, AKR1E2, ENSECAT00000061257.2, ENSECAT00000063563.1, ERICH4, ENSECAT00000067615.1, RANBP3L
eca-miR-628a	ENSECAT00000015056.3, FGF6, SVOP, BCL11B, CDK17, CHD1L, ENSECAT00000047574.1, ENSECAT00000047991.2, CEMIP, ENSECAT00000052831.2, ZNF577, HAO1, NTN4, VPS37C, RSPRY1, SLC22A13, LSP1, SAMM50, LRRFIP2, MEPE, CDKL3, GEMIN2, ENSECAT00000037036.1, TCF7L2, TUFM, ENSECAT00000073592.1, ENSECAT00000073972.1, ENSECAT00000078711.1, NR1D2, PTPN5, TENT2, SAA1, CXHXorf58, DTNB, LGALSL, RNMT, PTBP1, SLC22A2, ENSECAT00000023632.3, SNX9, ENSECAT00000037681.2, ENSECAT00000071821.1, PRMT7, LRRC18, NCOA4, PTPN20, ASAH2, RNF121, GPNMB, ENSECAT00000061755.1, ENSECAT00000067029.1, ENSECAT00000068718.1, ENSECAT00000070128.1, ENSECAT00000070917.1, ENSECAT00000075732.1, ENSECAT00000078060.1, MORC4
eca-miR-652	MCCC1, GLI4, LHX8, ENSECAT00000053245.2, ZNF668, XXYLT1, LIAS, MYOG, VDR, TUB, ENSECAT00000020753.2, ENSECAT00000020779.2, RNF186, SUMF1, EXOC7, ENSECAT00000042764.1, ENSECAT00000046422.1, ZNF135, ERGIC1, KRT82, LSS, ATP6V0D2, TRIM14, PIGU, ZDHHC14, MKS1, ENSECAT00000043659.2, ENSECAT00000056509.2, POLR1G, ENSECAT00000021769.3, ENSECAT00000076265.1
eca-miR-7	ZNF605, CEP57L1, EGF, MRPL24, ENSECAT00000015278.3, MMD2, TRIM35, CCNA1, AKAP9, ENSECAT00000022488.3, STAC, NDUFA8, DENND4A, PKP4, FGGY, TDRD3, ENSECAT00000035498.1, IL12RB2, ENSECAT00000041266.2, ENSECAT00000045157.2, SEPTIN9, ENSECAT00000054356.1, FAM221B, OR6B6, NCR1, SAXO1, PPWD1,

	SH2D6, ANKRD55, LRCOL1, UBASH3A, TMEM216, HTR3A, THEM5, MYOT, NDUFA10, FDXR, ENSECAT00000052633.2, ENSECAT00000058452.1, ENSECAT00000001006.2, OR7R4, S1PR3, ACSM3, KBTBD8, SLC27A6, CPEB2, AUH, RNF217, ACBD5, FNDC8, DPAGT1, JPH1, INSYN2B, CDKN3, DKK3, CCNQ, MOB3A, ENSECAT00000034978.1, RFFL, ENSECAT00000048810.1, ZNF746, CYTH4, ENSECAT00000062388.1, WDR61, LRRC36, PITPNC1, POMGNT2, TGM7, MATN2, GPC3, B4GALNT1, LRGUK, GYS1, NAPB, FARP2, LSS, RYK, DLC1, PFKP, ENSECAT00000054068.2, DPP9, ADAM12, PLPP1, SCAP, ENSECAT00000061529.1, ACTR1B, ABCC5, HAUS1, TMEM214, PRDX1, SDS
eca-miR-744	RBM38, DRAM1, ARHGAP29, PERP, PRRG1, RHPN2, CLMN, SYPL2, TRMT44, DBX2, PLPPR2, ITM2B, NSUN3, ANO4, PPP1R16A, PPL, SLC25A26, TMPO, FBXL4, PLA2G12A, PIK3C2B, GATA2, GINM1, TRMT11, FSTL4, SRP54, CD99L2, ECRG4, DLX6, SRSF12, MED24, CLTC, RGMA, CAV3, DUSP8, ROCK1, GTF2F2, ENSECAT00000024753.3, MDH2, CDK20, SNX31, METRNL, CEP85L, ENSECAT00000041145.2, MFSD9, BCL11B, MYO18A, GOLM1, TCF7, ENSECAT00000000623.2, OR7D19, ENSECAT00000002171.2, BEST1, MINDY1, PINK1, LRRC28, DBN1, FAM118A, SLC22A8, HES7, ENSECAT00000015797.2, DOC2A, KDSR, RNF186, ENSECAT00000022111.2, SQSTM1, NKX2-1, IRX3, B3GNT8, NDUFA10, ENSECAT00000032732.1, FBXO9, NPRL3, CLASRP, CRIP2, ENSECAT00000052482.1, ENSECAT00000052597.1, MAZ, OTOP3, GNG5, ENSECAT00000062152.1, OR7D20, ENSECAT00000077804.1, NIPA1, ENSECAT00000001806.2, ENSECAT00000005898.2, TESPA1, MPZL3, ENSECAT00000011210.3, SYNPR, ARID3A, KIAA2013, EPN2, SPATA21, ENSECAT00000019268.2, PTBP1, DEPTOR, RRAS2, SERPINE3, CXCR5, ENSECAT00000045973.2, ID1, SLC25A29, ENSECAT00000067646.1, ENSECAT00000072366.1, H2BW4, ENSECAT00000074957.1, LRRC20, GHITM, TCL1A, PTCH1, CSF1R, IHH, APBB3, ENSECAT00000006703.3, SLC25A14, ENSECAT00000042822.1, MARK4, BAIAP2, PIP4K2B, CLCN7, HDGFL2, MTHFR, NUDCD3, FOXP4, SAMD11, PLIN4, REEP6, BSG, COL27A1, ENSECAT00000066102.1, SIX5, PGLS, DIAPH1, PRPF6, TTYH3, ENSECAT00000071629.1, ENSECAT00000076974.1, ENSECAT00000078109.1, SIDT2
eca-miR-872	TIAL1, ENSECAT00000022069.2, SERPINA5, ENSECAT00000033688.1, ADGRV1, SLC35A5, PNO1, ZFP92, TAT, ACER3, SKP2, CELF2, ZDHHC7, ENSECAT00000053052.2, IL13RA2, ENSECAT00000055011.1, ENSECAT00000061988.1
eca-miR-889	CXADR, EMX2
eca-miR-92b	DERPC, MBTPS1, ANO4, LMO7, ENSECAT00000039404.2, GPC4, TTC39B, UFM1, SETD3, DYNLT1, ENSECAT00000017008.3, ENC1, SLC30A4, SOAT2, ENSECAT00000023640.3, LCP1, ENSECAT00000033931.2, ENSECAT00000067669.1, ENSECAT00000075569.1, ENSECAT00000078698.1, SLCO2A1, TTC7A, ENSECAT00000070759.1
eca-miR-95	ST3GAL1, LYSMD2, ENSECAT00000018529.2, PTDSS2, ENSECAT00000033075.2, ENSECAT00000043200.2, ENSECAT00000064231.2, ENSECAT00000074094.1, NPL, MPP7, ENSECAT00000021967.3, ENSECAT00000024982.2, ENSECAT00000039027.1, ENSECAT00000044823.2, ENSECAT00000048251.1, ENSECAT00000056427.2, PBX1
eca-miR-96	NLRC5, GPT2, NR2C1, GYG2, DBN1, ABRA, SURF2, ZNF317, MAD2L1BP, OXSM, ENSECAT00000007999.2, TWSG1, LRRC7, ENSECAT00000033885.2, MAS1, ENSECAT00000049396.2, PTPN14, PGBD5, CHRM3, XIRP2, PAQR7, RNF121, MAF, METTL21A, ICAM1, RGL1

eca-miR-98	AASDH, EGR2, SAA1, DBX2, KRBA2, ZCCHC24, MYOM2, VASH1, ODF2L, CD69, PEBP4, SLC2A9, GPHN, ENSECAT00000034534.2, MARVELD2, TSPAN19, TACC2, PRUNE1, ENSECAT00000001706.2, CA14, ENSECAT00000005662.2, GYG2, ENSECAT00000007785.2, BRS3, ENSECAT00000009402.2, ENSECAT00000010519.3, DEAF1, ENSECAT00000014334.3, LRRIQ3, ENSECAT00000015604.2, YIPF3, MCAT, ENSECAT00000021387.3, ENSECAT00000021796.3, ADGRG5, USH1G, ALG12, SLC22A18, FAM166B, ENSECAT00000031001.1, ENSECAT00000031186.2, ENSECAT00000031256.2, MUC21, SEC24A, ENSECAT00000035002.2, ENSECAT00000036131.2, ADAMTS6, ENSECAT00000036347.1, ENSECAT00000037155.2, ENSECAT00000039175.2, KLHL5, ENSECAT00000042267.2, ENSECAT00000043787.1, ENSECAT00000047741.1, DNAJB4, ENSECAT00000049845.1, ENSECAT00000052621.1, ENSECAT00000056314.2, ENSECAT00000056938.2, GPR27, DCST1, CDK17, ENSECAT00000064540.1, ENSECAT00000064869.1, ENSECAT00000066791.1, GALC, ENSECAT00000071372.1, ENSECAT00000073432.1, ENSECAT00000075304.1, ENSECAT00000076085.1, ENSECAT00000076118.1, GNB5, TMEM86B, ENSECAT00000077733.1, ENSECAT00000078240.1, GAD2, ENSECAT00000000774.2, ENSECAT00000002355.3, F2RL1, SLC44A1, ENSECAT00000005033.2, MFSD8, KCNRG, TSPAN6, SERINC4, IL34, TENT2, NAPG, SKP2, TAF7L, NFATC3, LOX, PLXNB3, RHBDL2, FOXR1, ZNF324, CCKAR, CACNG2, GPR142, GCNT1, GPR50, CCNJL, ENSECAT00000057286.1, TLE2, ENSECAT00000064290.2, ENSECAT00000076737.1, MASP2, POMGNT2, OIT3, CCR2, LY6D, ARHGEF28, HM13, PLEKHG3, FAF1, ENSECAT00000046247.1, CHP2, FBN1, GTPBP8, TUBB, ASAP1, GALNT15, CRYBG2, ASPH, ZNF692, BZW1, OPHN1, NDUFA9, TMEM52B, NFKBIB
eca-miR-9a	WNT10B, ZNF605, TYRP1, TMPO, IDH3A, ENSECAT00000017767.3, DMRTB1, PCSK1, CALML4, BZW2, ESPL1, SLC16A7, BSDC1, MRLN, GLT8D2, ABHD12, ENSECAT00000000577.2, ENSECAT00000007785.2, PGM2, SENP1, PROSER1, THOC5, GLB1, MHCB3, SUGP1, CAPG, SNCA, ENSECAT00000017022.2, STOML2, RABGAP1L, TIMM17A, HABP2, MBTD1, ENSECAT00000030253.2, ENSECAT00000032034.1, FBXO9, TNNT2, ENSECAT00000036699.2, EQMHCC1, DEUP1, ENSECAT00000042285.1, ENSECAT00000043535.2, TRAF3IP1, ENSECAT00000057076.1, ENSECAT00000059848.2, ENSECAT00000061427.1, ENSECAT00000062741.2, ENSECAT00000063846.2, GALP, ENSECAT00000067367.1, ENSECAT00000068639.1, ENSECAT00000071372.1, ENSECAT00000073811.1, ENSECAT00000074810.1, ENSECAT00000077045.1, RAPGEF5, PRKAR1A, HSPB3, ACTL7A, RDH10, HYKK, ENSECAT00000007697.2, SFXN5, AUH, TENT2, TBCEL, PRSS58, SHC4, CTH, ABI1, PRR16, CLMP, MLEC, NPAS1, B3GAT2, PSMC5, SPIN1, KCNS3, SLC39A11, DHRS7B, C2H1orf94, ENSECAT00000049647.1, ENSECAT00000061560.2, ENSECAT00000063860.2, ENSECAT00000078366.1, RUFY2, ENSECAT00000029905.2, ENSECAT00000071185.1, ENSECAT00000043089.2, WFS1, RAB11FIP4, FCHO2, KCNC4, PLCL2, TBC1D9B, ENSECAT00000047518.1, MFSD5, BCAN, VPS13A, FGF4, ACO1, KIF23, PECAM1, FAM170B, AFTPH, ENSECAT00000065056.1, HSD3B2, GPR83, XKR8, DIAPH1, TTYH3, RAP1B
novel-eca-miR-13	PARP6, NHLRC3, PIGQ, ARSL, MIER3, KIAA0930, ENSECAT00000045826.1, ZDHHC6, CAPN5, TBC1D20, COPS3, ENSECAT00000046430.2, NIPA2, OLFM1

novel-eca-miR-14	INF2, TKFC, ENSECAT00000018919.2, CD244, CHRM1, SH3BGR, STX4, ENSECAT00000014709.2, ERCC8, BSCL2, PLA2G2C, SLC30A6, TH, CERK, PARVB, TYMP, ALOX15B, LMO4, OR4F1, ENSECAT00000062563.1, ENSECAT00000066672.1, ENSECAT00000073343.1, SLC27A6, PLEKHA2, ZNF189, PDZD9, ELF2, PSMA1, DPYSL5, NINJ2, USP30, PRMT7, TMEM86A, C31H6orf118, ARHGEF10L, ZC3H12C, ENSECAT00000044543.1, B4GALNT2, CAMK2A, RC3H1, PSMD12, EPHA6, ERCC6L2, R3HCC1, ENSECAT00000056993.1, UBXN6, ENSECAT00000063223.1, R3HCC1L
novel-eca-miR-25	USP39, MRPL39, UBE2A, SLC35F1
novel-eca-miR-35	ENSECAT00000014060.3, ENSECAT00000015375.3, ENSECAT00000016839.3, PSME2, MTFR1, EXTL3, SERPINE1, ALG13, AMMECR1L, MARCKS, ENSECAT00000037475.2, CDK17, CCDC18, ENSECAT00000043387.2, RASGEF1B, HHLA2, KCNJ6, OR2Z1, GUCY1A1, SHTN1, SUSD5, F12, MED17, MCRS1, POLR1E, LSP1, MPL, ENSECAT00000018689.2, ASB2, CTDSP2, CNDP1, PGS1, CDK3, ENSECAT00000023424.2, ENSECAT00000023427.3, FBP1, CD19, ENSECAT00000031306.2, CD6, FOXP1, NSMCE1, ENSECAT00000040109.1, ENSECAT00000041303.2, CMTM6, ENSECAT00000059217.1, BPIFA2, SULT4A1, ELMOD3, INPP5E, SRGAP3, VDAC3, DDI2, ENSECAT00000001505.2, ENSECAT00000005071.3, PHGDH, CDK14, IPMK, GAREM2, KCNRG, SLC5A6, GABRE, C28H12orf42, ATP6V0D1, ENSECAT00000018064.2, TBX5, USP30, STK25, D2HGDH, PAK1, PPP3R1, CDADC1, SSTR2, ENSECAT00000023212.2, SLC22A5, ENSECAT00000023632.3, BPIFB2, TCHP, GULP1, MINAR2, BCLAF3, GATA6, ENSECAT00000032312.2, ENSECAT00000035857.2, BGN, ENSECAT00000037681.2, LMNB2, PRIM2, DPYSL5, ENSECAT00000053505.2, ENSECAT00000057352.2, TAC1, LRRC3B, ENSECAT00000071821.1, ENSECAT00000074784.1, FNDC1, CHRM3, TMEM26, LDB3, FADS2, HAP1, FHOD3, ANK2, CUX1, FAM91A1, HSPA12A, FGFRL1, ENSECAT00000053644.2, RUSF1, RABEPK, PARD3B, CD55, CELSR2, ENSECAT00000064381.2, KLHDC8A, PRMT5, RAB31, LALBA, PPARD, HBS1L, ARHGAP30
novel-eca-miR-42	KANK4, GUCY1A1, PHF10, MKRN1, RIBC2, ENSECAT00000005519.2, ENSECAT00000008190.2, IL34, ABHD3, ST6GALNAC2
novel-eca-miR-44	LRRTM4, CNTNAP1, MTFR1, ENSECAT00000035436.2, SULT4A1, PPP2R3C, EDIL3, OR5P5, CPNE2, MYO15B, EQUCABV1R924, TGFBR2, FAM172A, ENSECAT00000044095.1, MCOLN2, OR4G27, NRXN3, AQP10, LMAN1, ENSECAT00000069854.1, KCNK10, CD44, CRYM, FAM151B, SERBP1, GLA, RAB9B, ALG8, ARMCX2, CD96, C2H1orf94, OR13D2G, SNTA1, MARVELD3, CTH, SLC12A6, PTPN1, CFAP45, SP110
novel-eca-miR-49	CD226, DUSP10, GRIK3, TRIML2, MEGF6, REPS1, ITIH2, EDAR, WDR26, ADGRL1, ENSECAT00000030286.2, ENSECAT00000030791.2, CENPL, ENSECAT00000032960.2, CSF3R, FAM189A2, ENSECAT00000048265.2, ENSECAT00000049023.2, FAM221B, C1QL4, ENSECAT00000006837.3, ZNF668, RSPH1, PNLIPRP1, NPRL3, PDE9A, LSG1, DMRTC2, ENSECAT00000019643.2, KLF3, ENSECAT00000023415.2, PTK7, TMC6, CCDC184, CYP2S1, ENSECAT00000043523.2, GRAMD4, ENSECAT00000064382.1, EQMHCC1, ENSECAT00000068433.1, ZFYVE27, ISL1, ENSECAT00000005058.2, CYP46A1, MIDN, FAM71A, ALG2, VEGFD, SLC47A1, PLEKHA2, ILDR1, ALKBH2, REPIN1, ANKRD42, RBFOX2, TRIB1, IDO2, LCAT, UBOX5, NECTIN3, CD80, FRMD6, ENSECAT00000044119.2, NDRG1, DUSP9, CBY2, ENSECAT00000062545.1, ENSECAT00000064055.1, PGRMC2, ENSECAT00000072717.1, COA6,

	SLC35F3, TYSND1, MYOZ1, ENSECAT00000056949.2, ENSECAT00000057973.2, ABCC5, SLC6A11, ENSECAT00000042908.2, ATG9B, TNS1, ABL1, PLXNB3, DTX4, WNK1, MTG2, KHNYN, GLUL, JAKMIP1, MON1B, ANO8, LCN9, PTPA, ENSECAT00000059913.2, STAC, FXYD6, KRT1, DTNA, CHRD, SMO, ENSECAT00000074178.1, ATG13, TMEM98, ENSECAT00000075259.1, APPL2, LZTS3, INPP5J, ENSECAT00000078353.1
